# Legacy effects of fumigation on soil bacterial and fungal communities and their response to metam sodium application

**DOI:** 10.1186/s40793-022-00454-w

**Published:** 2022-12-03

**Authors:** Xiaoping Li, Victoria Skillman, Jeremiah Dung, Kenneth Frost

**Affiliations:** 1 Virginia Tech, Hampton Roads Agricultural Research and Extension Center, Virginia Beach, VA 23455 USA; 2 Hermiston Agricultural Research and Extension Center, Oregon State University, Hermiston, OR 97838 USA; 3 Central Oregon Agricultural Research and Extension Center, Oregon State University, Madras, OR 97741 USA; 4 Department of Botany and Plant Pathology, Oregon State University, Corvallis, OR 97333 USA

**Keywords:** Soil microbiome, Soil health, Fumigation, Metam sodium, Disease management, Sustainable agriculture, DNA metabarcoding, Illumina MiSeq

## Abstract

**Background:**

Soil microorganisms are integral to maintaining soil health and crop productivity, but fumigation used to suppress soilborne diseases may affect soil microbiota. Currently, little is known about the legacy effects of soil fumigation on soil microbial communities and their response to fumigation at the production scale. Here, 16S rRNA gene and internal transcribed spacer amplicon sequencing was used to characterize the bacterial and fungal communities in soils from intensively managed crop fields with and without previous exposure to metam sodium (MS) fumigation. The effect of fumigation history, soil series, and rotation crop diversity on microbial community variation was estimated and the response of the soil microbiome to MS application in an open microcosm system was documented.

**Results:**

We found that previous MS fumigation reduced soil bacterial diversity but did not affect microbial richness and fungal diversity. Fumigation history, soil series, and rotation crop diversity were the main contributors to the variation in microbial β-diversity. Between fumigated and non-fumigated soils, predominant bacterial and fungal taxa were similar; however, their relative abundance varied with fumigation history. In particular, the abundance of Basidiomycete yeasts was decreased in fumigated soils. MS fumigation also altered soil bacterial and fungal co-occurrence network structure and associations. In microcosms, application of MS reduced soil microbial richness and bacterial diversity. Soil microbial β-diversity was also affected but microbial communities of the microcosm soils were always similar to that of the field soils used to establish the microcosms. MS application also induced changes in relative abundance of several predominant bacterial and fungal genera based on a soil’s previous fumigation exposure.

**Conclusions:**

The legacy effects of MS fumigation are more pronounced on soil bacterial diversity, β-diversity and networks. Repeated fumigant applications shift soil microbial compositions and may contribute to differential MS sensitivity among soil microorganisms. Following MS application, microbial richness and bacterial diversity decreases, but microbial β-diversity was similar to that of the field soils used to establish the microcosms in the short-term (< 6 weeks). The responses of soil microbiome to MS fumigation are context dependent and rely on abiotic, biotic, and agricultural management practices.

**Supplementary Information:**

The online version contains supplementary material available at 10.1186/s40793-022-00454-w.

## Background

Soil microorganisms are an essential component of healthy soils and plants. They directly and indirectly contribute to the pivotal soil processes and functions, including decomposing organic matter, transforming and cycling carbon, fixing nitrogen, and maintaining soil structure [1]. They also play an important role in maintaining plant health and productivity. For example, some *Streptomyces* strains suppress potato scab disease [2] and some *Bacillus* strains can induce plant systemic resistance to bacterial leaf blight in rice [3]. Additionally, many soil microorganisms can promote plant growth by secreting plant growth hormones, solubilizing phosphate, or fixing nitrogen [4]. However, intensified management practices of farming, high agrochemical inputs and simplified cropping systems, can result in legacy effects on the soil microbiome [5–8] that may indicate changes to soil function [9–11]. In order to enhance plant fitness and productivity, it is necessary to understand how these varied and repeated crop management activities can be used to optimize soil and plant microbiome.

Soil fumigation is a common management practice used to suppress soilborne plant pathogens in contemporary faming systems [12, 13]. Many soil fumigants are thought to have a broad spectrum of activity, suppressing target and non-target (e.g., free-living or beneficial) microorganisms. Some negative consequences associated with fumigation have been documented, including reduced soil biomass, soil enzyme activities, and carbon/nitrogen cycling and mineralization [12, 14–16]. Repeated use of fumigation also has legacy effects on the soil microbial communities. For example, Zhang et al. [17] documented that repeated soil fumigation with chloropicrin decreased microbial richness and diversity but increased the abundance of *Actinobacteria* and *Saccharibacteria*. Additionally, Dangi et al. [18] demonstrated that microbial communities of non-fumigated soils were different from microbial communities of soils fumigated with methyl bromide and/or chloropicrin. These shifts in soil microbiome may cause dysbiosis in the soil, leading to opportunities for invasion or recolonization of soilborne plant pathogens that were not present or problematic prior to fumigation [19–21]. Consequently, changes to the soil microbial community that occur as a result of soil fumigation may be indicative of changes to important soil processes or functions that can influence agricultural productivity [22]. For that reason, knowing the long-term impacts of fumigation on the soil microbial diversity, structure, and function may help improve the sustainability of farming operations [23].

Sodium N-methyldithiocarbamate, or metam sodium (MS), is a routinely applied soil fumigant within many cropping systems that is used to control weeds, nematodes, and soilborne diseases [24]. After application, MS quickly hydrolyzes in the soil to produce the active biocidal compound methyl isothiocyanate (MITC) [25], which is considered to have broad spectrum antimicrobial activity [12, 26]. The fate of MITC is the result of MITC generation and dissipation rates. In soil, MITC production peaks around 6 h after MS application and dissipation from the soils varies from 2 to over 6 days depending on the soil type [27]. In general, disease control efficacy correlates with the MITC exposure, calculated as the product of MITC concentration and time [27–29], although measurement of MITC exposure in field soils is challenging.

MS fumigation has primarily been studied for the management of specific pests or diseases and there are relatively few studies that have examined its effects on the non-target organisms or the soil microbiome. Toyota et al. [30] documented a non-lasting (i.e., 26-days) effect of MS on total and culturable bacterial population with an initial suppressive effect. Corden and Young reported [31] that MS temporarily decreased fungal population while others showed mycorrhizal fungi were particularly sensitive to MS [32, 33]. Recent studies with high-throughput sequencing have shown MS negatively affected the diversity and richness of soil bacterial communities and also altered the bacterial compositions [34, 35]. Several recent laboratory microcosm studies have tried to examine how MS or dazomet fumigation affects soil bacterial β-diversity and/or function [35–37]. In general, all the studies reported the changes of soil bacterial community diversity and structure [35–37]. Despite the widespread use of MS fumigation to manage soilborne pests and diseases in multiple cropping systems, there is a lack of culture-free studies that have evaluated the impacts of MS on soil fungal communities or examined how the soil microbiome responds to MS applications to soil from commercial farms or in a field setting.

In this study, we used DNA metabarcoding with high-throughput amplicon sequencing to examine how the bacterial and fungal communities might be affected by a soil’s previous MS exposure history. The objectives of this study were to (1) characterize how the soil microbiome varies as a function of MS exposure history and other abiotic factors (i.e., rotation crop diversity, soil series, soil pH) and (2) examine how the response of the soil microbiome to MS application differs depending on previous exposure to MS, a fumigant commonly used in Pacific Northwest potato cropping system.

## Methods

### Farm description and soil sampling

In 2018, between February 27 and March 20, soils were sampled from 13 fields, including 12 fields located in Oregon and one field located in Washington (Table 1). All fields were located within the irrigated crop production region known as the Columbia Basin. Fields in Oregon were located in the Columbia River Basin, near Boardman, Oregon. The field in Washington was located approximately 12.9 km south of Moses Lake. In Oregon, fields were selected based on their previous management history, obtained prior to sampling, and to maximize geographic distances between sampled fields within a farm. Based on the available management histories, these fields varied in their previous exposure to MS fumigation, ranging from never fumigated to five fumigation events (Table 1). Soils were sampled from six fields that had never been fumigated and seven fields previously fumigated. Eleven fields were further divided into four quadrants, considered field replications, and two fields were divided into two quadrants, resulting in 48 sample locations that were georeferenced prior to sampling. For each quadrant, soil cores (N = 15) to the depth of approximately 40.6 cm were collected in a zig-zag pattern along a 10 m transect from the field edge to the field center. Soil cores were pooled into a collection bag and mixed well by shaking and turning the collection bag upside down 10 times. Ten grams of each soil was placed into a 15 ml centrifuge tube (Falcon® by Corning Inc. New York, USA). Centrifuge tubes were placed on dry ice for transport to the laboratory where they were stored at − 20 °C until DNA extractions occurred. The remaining soils were placed in coolers on ice for transport to the laboratory and stored at 4℃ in the laboratory prior to conducting assays to determine the presence and abundance of soilborne pathogens. At each sample location, an additional 75 L of soil was collected with a shovel to a depth of 25 cm for microcosm establishment.

**Table 1:** Descriptive information for each field soil in this study

Fields^a^	Reps	Cropping system	pH	Soil series	Soil type	Rotation crop diversity	Dominant crop (# of years)	Metam Sodium Applied (# applications)^c^
f1	4	Conventional	7.33	Koehler/Quincy	Loamy sand/Fine sand	6	Potato (7)	1141 (3)
f2	2	Conventional	7.32	Koehler	Loamy sand	6	Corn (7)	1506 (4)
f3	4	Conventional	7.29	Koehler/Taunton	Loamy sand/Fine sandy loam	5	Alfalfa (11)	0 (0)
f4	4	Conventional	6.87	Koehler	Loamy sand	6	Potato (7)	1421 (4)
f5	4	Conventional	7.05	Koehler/Quincy	Loamy sand/Fine sand	6	Potato (7)	1777 (5)
f6	4	Conventional	6.91	Quincy	Fine sand	4	Corn (7)	702 (2)
f7	4	Conventional	6.98	Quincy	Fine sand	5	Potato (7)	1122 (3)
F8	4	Conventional	7.08	Quincy	Fine sand	4	Potato (7)	1216 (3)
f13^b^	4	Conventional	5.24	Timmerman	Sandy loam	4	Wheat (8)	0 (0)
f9	2	Organic	7.02	Sagehill/Taunton	Very fine sandy loam/Fine sandy loam	2	Corn (4)	0 (0)
f10	4	Organic	7.13	Quincy	Fine sand	7	Corn (4)	0 (0)
f11	4	Organic	7.46	Koehler/Sagehill/Taunton	Loamy sand/Very fine sandy loam/Fine sandy loam	4	Corn (5)	0 (0)
f12	4	Organic	7.25	Quincy	Fine sand	3	Corn (3)	0 (0)

^a^Fields f1–f6 had 21 years of crop rotation history; f7 had 20 years; f8 and f13 had 19 years; f10 and f11 had 13 years; f9 had 7 years, and f12 had 6 years

^b^Amended with mustard green manure

^c^Liter per hectare

### Quantifying field cropping history

Each field’s cropping history was obtained from our grower cooperators and two aspects of management history were quantified: 1) Rotation crop diversity, calculated as the total number of unique crop species in the rotation (Tables 1, 2) Fumigation history, a categorical variable based on MS application history (i.e., fumigated, not fumigated).

**Table 2:** PERMANOVA showing each model term in describing variation in microbial β-diversity of farm soils

Variable	Bacteria				Fungi			
	PERMANOVA		Dispersion		PERMANOVA		Dispersion	
	*R*^2^	*P-*value	Pseudo-*F*	*P-*value	*R*^2^	*P-*value	Pseudo-*F*	*P-*value
Fumigation history	0.102	** < 0.0001**	6.86	0.0098	0.087	** < 0.0001**	1.01	0.3200
Soil series	0.306	** < 0.0001**	2.57	0.0591	0.139	**0.0011**	3.26	**0.0223**
Crop diversity	0.055	** < 0.0001**	–^a^	–	0.033	**0.0349**	–	–
Soil pH	0.010	0.6452	–	–	0.015	0.6612		–

^a^Not tested

*P-*values less than 0.05 are in bold values

### Soil characteristics

Using the GPS coordinates of each sample location, soil series metadata was collected using a customized R script that implements soil tool kits developed by members of the National Cooperative Soil Survey (NCSS) [38–43]. Briefly, a 15 × 15 m^2^ box was defined for each sample location and soil taxonomy metadata were retrieved from the database for each sample area and amended to our data set. The type of the top 25 cm soils was retrieved according to the corresponding soil series from the USDA Official Soil Series Descriptions and Series website [44]. pH was directly measured for each soil sample. Briefly, five grams of soil was suspended in 5 mL of distilled water and the mixture was left to rest for 5 min. Two readings were taken for each sample on an Orion pH meter (Model 410A, Thermo Scientific, Waltham, MA, USA) and the average was calculated (Table 1).

### Microcosm experiment

Soil columns (N = 48) were constructed from 30.5 cm diameter PVC pipes cut to a length of one meter. A shorter PVC cylinder with a piece of landscaping cloth stretched over its top was placed in the bottom of each column so that soils placed in each column would remain in the column and not contact field soils at the bottom of the column. The landscape cloth also allowed for water to drain from the bottom of each column. On March 1 and 2 of 2018, columns were buried 0.75 m deep in the ground with the top of the column open to the environment. This allowed for filling, sampling, fumigant application, and irrigation. Soils from each field location were placed into the PVC columns on March 22; approximately, 53 L of each field soil was placed into each column. Columns were allowed to equilibrate for 21 days after filling and placement in the environment.

On April 12, 2018, columns were sampled prior to MS fumigation. Briefly, soils were sampled with a 1.3 × 17.8 cm turf soil corer (AMS, American Falls, ID, USA), and three cores per column were collected at depths of 10 cm, 20 cm, and 30 cm. The three soil cores taken at each depth were combined, homogenized, placed in a plastic bag, transported on ice to the laboratory and stored at − 20 °C until DNA extractions occurred. After sampling on April 12, columns were wetted with approximately 1 L of water to ensure soil moisture was appropriate for fumigant application. On April 13, 2.6 ml of Vapam HL (Active ingredient: 42% sodium N-methyldithiocarbamate) was mixed with 1304 ml water and applied to each column to simulate a chemigation application of Vapam HL at a rate of 374 L per hectare applied in 1.9 cm of water per unit area. Columns were sprinkler irrigated approximately every 3 days with 0.64–0.76 cm of water. Soils were sampled at three depths as described above at one, three, and 6 weeks after fumigation. Germinating vegetation was removed from the columns during the sampling period.

### Soil DNA extraction, library preparation, and DNA sequencing

To prepare soils for DNA extraction, approximately 0.25 g of soil was weighed out, transferred to a PowerBead Tube of the DNeasy® PowerSoil® Pro Kit (Qiagen, Hilden, Germany), and DNA was extracted following the manufacturer’s instructions with minor modifications, including using a Mini-Beadbeater-24 (Bio Spec Products Inc., Bartlesville, OK, USA) to homogenize the soil mixture. DNA concentration and quality were assessed for each sample by examining absorbance 260/280 and 260/230 ratio as measured on a Nanodrop 2000 spectrophotometer (Thermo Fisher Scientific, Wilmington, DE, USA).

DNA samples were used as template for library preparation and Illumina sequencing. Briefly, primers targeting bacterial 16S rRNA gene V3–V4 region and fungal internal transcribed spacer region 2 (ITS2) were designed according to the guideline for Illumina MiSeq system. For 16S rRNA gene amplicon library preparation, we used primer pair 341F (CCTACGGGNGGCWGCAG) and 805R (GACTACHVGGGTATCTAATCC) [45] with Illumina overhang adapters. PCR reactions were carried out in a T100 thermocycler (Bio-Rad Laboratories Inc., Hercules, CA, USA). The PCR conditions for 16S rRNA gene amplicon amplification were: initial denaturing at 95 °C for 3 min, followed by 25 cycles of 95 °C for 30 s, 55 °C for 30 s, and 72 °C for 30 s and a final extension step of 72 °C for 5 min. For ITS2 amplicon library preparation, the forward primer ITS86F (GTGAATCATCGAATCTTTGAA) and reverse primer ITS4 (TCCTCCGCTTATTGATATGC) [46] with Illumina overhang adapters were used. The PCR conditions for amplification of ITS2 amplicon were an initial denaturation at 94 °C for 3 min followed by 30 cycles of 94 °C for 45 s, then 55 °C for 45 s, and 72 °C for 2 min and a final extension was at 72 °C for 7 min. PCR products were purified using the Wizard® SV gel and PCR clean-up system (Promega Corporation, Madison, WI) then shipped to Oregon State University Center for Quantitative Life Sciences for indexing PCR and Illumina Miseq sequencing (paired-end 300 bp). Raw sequencing reads were deposited in the National Center for Biotechnology Information Sequence Read Archive (NCBI SRA) database (BioProject: PRJNA688547). The data, metadata, and R scripts used for this study are available on the Github repository [47].

### Sequencing data analysis

The open-source platform QIIME2 (version 2019-07) [48] was used as our pipeline environment for sequence processing and analyses. Sequence qualities were assessed and then paired end sequences were merged, sequencing errors were corrected, sequences with chimera and singleton reads were removed, and sequences were de-replicated using the DADA2 [49] plugin in QIIME2.

#### Sequencing summary

From the field soils, there were 7,924,661 and 10,784,670 sequencing reads generated from the 16S rRNA gene and ITS2 libraries, respectively. After DADA2 denoising, the survival rates of the reads were 47% (3,477,548) for 16S rRNA gene and 65% (7,043,525) for ITS2. Further pruning removed taxa with fewer than 10 sequences and the amplicon sequence variants (ASVs) that were assigned to Archaea. The minimum sequencing depth was 39,275 for 16S rRNA gene and 29,042 for ITS2, which allowed all samples to be retained for analysis of bacterial and fungal communities. Overall, the 16S rRNA gene and ITS2 datasets contained 21,899 and 3028 ASVs assigned to 1241 and 481 bacterial and fungal genera, respectively.

From the microcosm soils, sequencing 16S rRNA gene and ITS2 spacer DNA libraries generated 36,543,720 and 41,782,352 raw reads, respectively. After denoising, 15,563,720 (42.59%) and 26,139,936 (62.56%) reads were retained in the 16S rRNA gene and ITS2 spacer DNA libraries, respectively. There were 21 samples with low numbers of sequences (< 7000) for the 16S rRNA gene library and 9 samples with low numbers of sequences (< 10,000) for the ITS2 library that were removed from the data set prior to analysis. After removal of Archaea and taxa with fewer than 10 sequences, there were 44,549 ASVs in 555 samples and 6063 ASVs in 567 samples in the 16S rRNA gene and ITS2 datasets, respectively.

Rarefaction curves were assessed for 16S rRNA gene and ITS2 sequences using the rarecurve function in the vegan package with 100 steps [50] (Additional File 1: Fig. S1). Sequencing depths of soils sampled from farm fields were adequate to retain all the samples in our analyses. However, for the microcosm experiment, only soil samples with more than 7000 sequences in the 16S rRNA gene library and 10,000 sequences in the ITS2 library were retained and used in our analyses. ASVs with total sequence counts less than 10 across all the samples were discarded. As a result, 19 and 6 of the 574 and 576 16S rRNA gene and ITS2 library samples were discarded, respectively.

Observed ASV richness and the inverse Simpson index were calculated using samples that were rarefied at depth of 39,275 and 29,042 sequences for bacterial and fungal communities in soils from farm fields, and at 7019 and 10,476 sequences for bacterial and fungal communities in soils from the microcosm experiment. Each index measurement was obtained without replacement using the rarefy_even_depth and the estimate_richness functions from the R phyloseq package [51].

#### Taxonomy classification

The optimized sequences were taxonomically classified using the SILVA database (release 132 for QIIME) [52, 53] curated with 99% sequence identity for bacterial identification, and the UNITE QIIME release (database version 8.0 release) for fungal identification [54, 55]. The V3-V4 region from 16S rRNA gene reference sequences and the full length ITS2 sequences were used to build naïve Bayes classifiers with the QIIME2 feature-classifier extract-reads plugin. The classifiers were then used to assign taxonomy using the DADA2 optimized sequences with the QIIME2 classify-sklearn plugin with a default confidence threshold of 0.7. A feature table containing counts of ASVs in each sample and a taxonomy table were exported in biom format [56] for statistical analysis and data visualization in R (version 4.1.2) [57]. Taxonomic rank was re-annotated to the higher and best-annotated rank assignment if an ASV was not unidentified or was annotated as: “uncultured”, “uncultured soil bacterium”, “uncultured bacterium”, “metagenome”, “wastewater metagenome”, “groundwater metagenome”, “uncultured organism”, “uncultured sediment bacterium”, “permafrost metagenome”, “uncultured forest bacterium”, and “microbial mat metagenome”.

### Statistical analysis

#### Microbial diversity and compositional analysis

##### α-diversity

For field soils, The Kruskal–Wallis test [58] was also used to assess the effect of fumigation history and soil series on measures of α-diversity. Dunn’s multiple comparison test [59] was used for pairwise comparison among the soil series if the Kruskal–Wallis test result was significant. A simple linear regression was used to assess the relationship between α-diversity and soil pH and crop diversity. The variation of α-diversity among each field was also examined with the Kruskal–Wallis test and Dunn’s multiple comparison test. *P*-values were adjusted using the Benjamini–Hochberg procedure [60]. Significance level was 5% for all statistical testing, including *P*-value correction until otherwise indicated.

For soils sampled from each microcosm, analysis of variance (ANOVA) was used to examine how measures of microbial α-diversity varied as a function of a soil’s fumigation history, sampling time, and the interaction between fumigation history and sampling time. The ANOVA assumptions were assessed using residual plots. A t-test was used to compare each time point (i.e., 1 week, 3 weeks, and 6 weeks) to the reference time (i.e., pre-treatment, or 0 week) using the rstatix package [61]. The *P*-values were adjusted using false positive rate (FDR) correction.

##### β-diversity

ASVs were grouped to the genus level and normalized by their relative abundance for β-diversity analysis [62]. Bray–Curtis dissimilarity [63] was used to estimate compositional dissimilarity of microbial communities among samples. Canonical analysis of principal coordinates (CAP) was used to examine correlations between environmental variables and β-diversity [64]. The capscale function of the vegan package was used to fit the full CAP model with covariates fumigation history, soil series, rotation crop diversity, and soil pH, which was then reduced to a parsimonious model using the ordistep function with 10,000 permutations and forward stepwise selection [50]. For analysis of data from microcosms, sampling time was added as a covariate to the model described above. Biplots were created in R to illustrate the ordination and association of the constrained variables to the microbial β-diversity. Analysis of variance (ANOVA) was used to determine the amount of variance described by the constrained variables and CAP axes. The adonis function [50] was used to conduct permutational multivariate analysis of variance (PERMANOVA) [65] with 10,000 permutations to quantify variation in centroid and spread attributable to the groups of interest (i.e., Fumigation history, soil series, etc.) and test hypotheses that there were no differences among the groups. Homogeneity of variances was assessed using the betadisper function of the vegan package with 10,000 permutations [50].

##### Biomarker identification and differential abundance analysis

Linear discriminant analysis Effect size (LEfSe) was used to identify genera that differed in abundance among fields with different fumigation exposure. The run_lefse function from the microbiomeMarker package was used to conduct the LEfSe analysis [66, 67].

For the microcosm study, the differentialTest function from the corncob package was used to evaluate change in abundance at each sampling time after MS application [68]. The sampling effect was evaluated separately based on the soil’s fumigation history and differential abundance of the 10 most predominant genera was evaluated using a likelihood ratio test (LRT). Ten thousand (n = 10,000) bootstrap iterations were performed for each test and *P-*values were adjusted using false discovery rate (FDR) correction. The R packages pheatmap [69] and ggplot2 [70] were used for visualizing the results.

### Co-occurrence network analysis

Network analysis and visualization of bacterial and fungal genera was performed on the field soils with different fumigation history using the R package NetComi [71]. The Sparse InversE Covariance estimation for Ecological Association and Statistical inference (SpiecEasi) algorithm [72] was used to construct the networks with parameters lambda.min.ratio set to 0.01, nlambda set to 20, and pulsar.params set to 50. Networks were constructed using 200 taxa with the highest frequency, this filtering resulted in the inclusion of 160 bacterial genera (11.5% of the total bacterial genera) and 149 fungal genera (31.0% of the total fungal genera). Multiplicative replacement (multRepl) was used for replacing zero values and a centered log-ratio transformation (clr) was used for normalization. A threshold of 0.6 was used to select edge connections between taxa pairs. The netAnalyze function was used to calculate network properties and centralities for all nodes were computed. Measurements of the degree, betweenness, closeness, and eigenvector were normalized. The nodes with the highest degree, betweenness, closeness, and eigenvector values were considered network hubs. Network comparisons were performed with 1000 permutations and *P-*values were adjusted using the Benjamini–Hochberg method [73]. The similarity of network clustering was compared based on the adjusted Rand index (ARI) [74] with the null hypothesis that the clusters from the two compared networks were not equal (H_0_: ARI = 0). The ARI value ranges from − 1 to 1, where 1 indicates two equal clusterings from the compared networks.

## Results

### Microbiome analysis of field soils

#### Microbiota diversity and richness

**Fig. 1:**
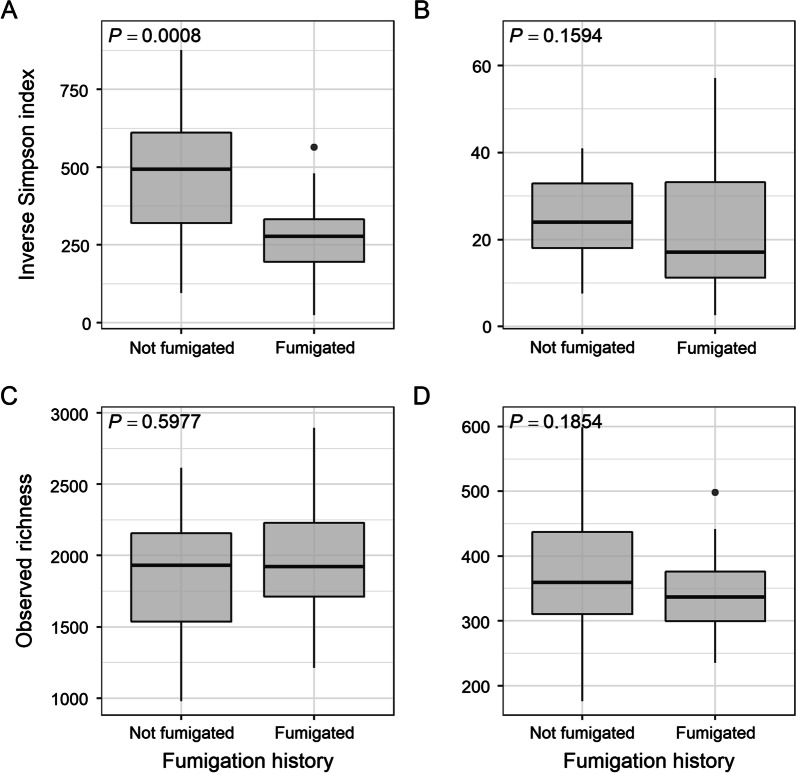
The inverse Simpson index and observed ASV richness of bacterial (**A** and **C**) and fungal (**B** and **D**) communities grouped by fumigation history. The overall *P-*value was calculated using the Kruskal–Wallis test

Fumigation exposure affected the diversity of the bacterial communities but not that of the fungal communities (Fig. 1). Bacterial diversity, as estimated by the inverse Simpson index, was lower (*P* = 0.0008) in fumigated soils than in non-fumigated soils (Fig. 1A). However, bacterial richness did not differ based on fumigation exposure (Fig. 1C).

Similarly, soil series influenced bacterial diversity and fungal richness, but not bacterial richness or fungal diversity (Additional File 1: Fig. S2). Specifically, fungal richness was greater in Timmerman soils than it was in Koehler and Quincy soils (Additional File 1: Fig. S2D). Bacterial diversity was the highest in Sagehill soils and lowest in Koehler soils (Additional File 1: Fig. S2A). However, no differences in bacterial diversity among soil series were identified by pairwise comparison even though the *P*-value of the overall Kruskal–Wallis test was less than 0.05 (Additional File 1: Fig. S2A). Additionally, rotation crop diversity (*R*^2^ = 0.32) and soil pH (*R*^2^ = 0.30) were negatively associated with bacterial diversity and fungal richness, respectively (Additional File 1: Fig. S3).

Diversity and richness of the bacterial communities varied among the fields but that of the fungal communities remained consistent (Additional File 1: Fig. S4). Comparatively, field 3 had lower bacterial diversity when compared to other non-fumigated soils (Additional File 1: Fig. S4A) and also had lowest bacterial richness among all fields (Additional File 1: Fig. S4C).

#### Microbial β-diversity in the field soils

**Fig. 2:**
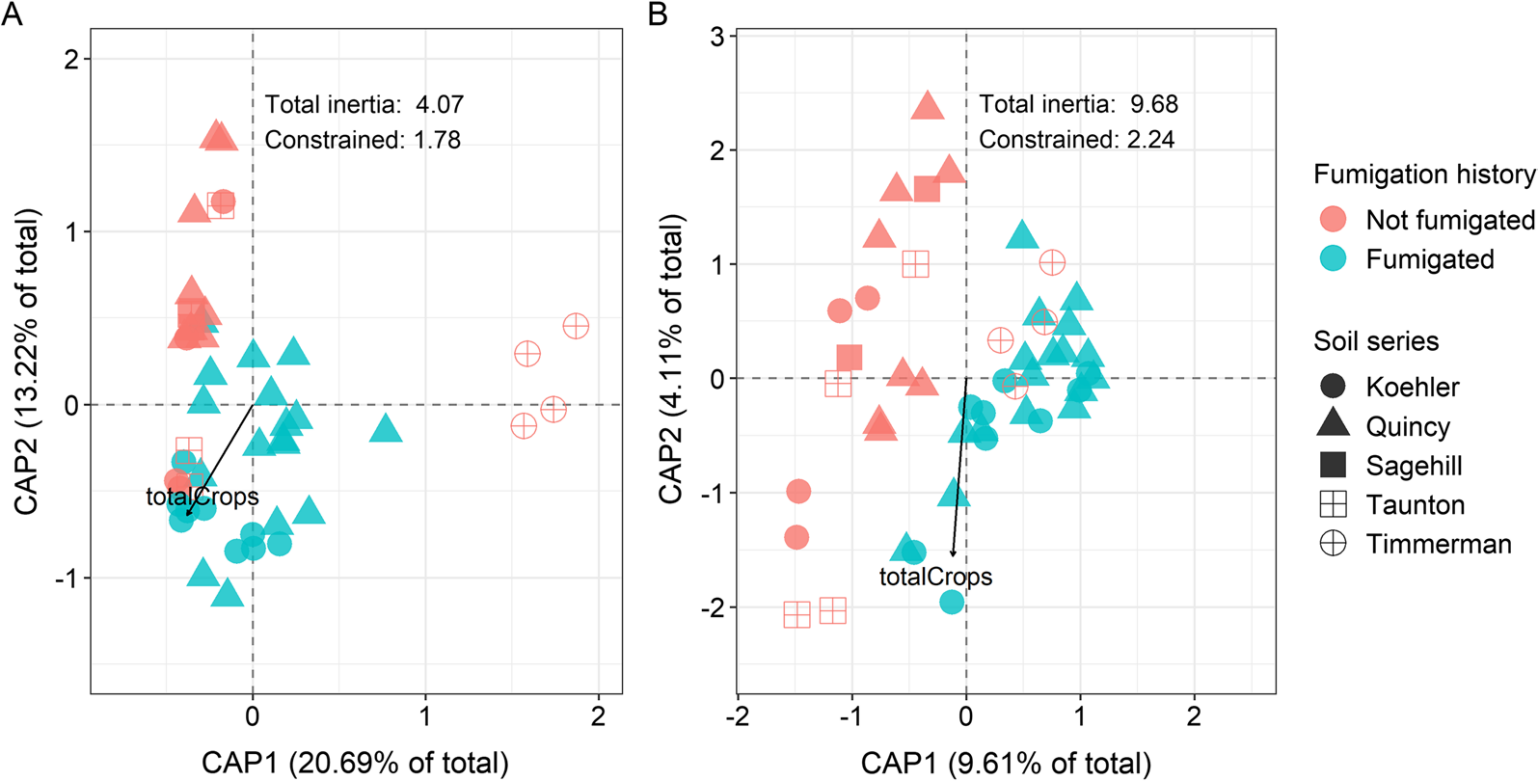
Bi-plot of the canonical analysis of principle coordinates (CAP) of the Bray–Curtis dissimilarity for bacterial (**A**) and fungal (**B**) communities. Fumigation history is indicated using color and soil series is indicated using shape. Arrows represent quantitative variables that point in the direction of increase. “totalCrop” is rotation crop diversity or the total number of crops in the field management history

Microbial β-diversity was influenced by fumigation history, soil series, and rotation crop diversity (Table 2). Based on a canonical analysis of principle coordinates (CAP), these three variables together accounted for 43.7% and 23.2% of the total variation in the bacterial and fungal β-diversity (Fig. 2A, B). Using permutational multivariate analysis of variance (PERMANOVA), fumigation history explained 10.2% and 8.7% of the total variation in bacterial and fungal β-diversity, respectively (Table 2), and soil series explained 30.6% and 13.9% of the variation in bacterial and fungal β-diversity. However, rotation crop diversity explained a smaller amount of variation in microbial β-diversity. Notably, bacterial samples classified by fumigation history and fungal samples classified by soil series were not homogeneously dispersed (Table 2).

#### Taxonomy composition and biomarker identification

**Fig. 3:**
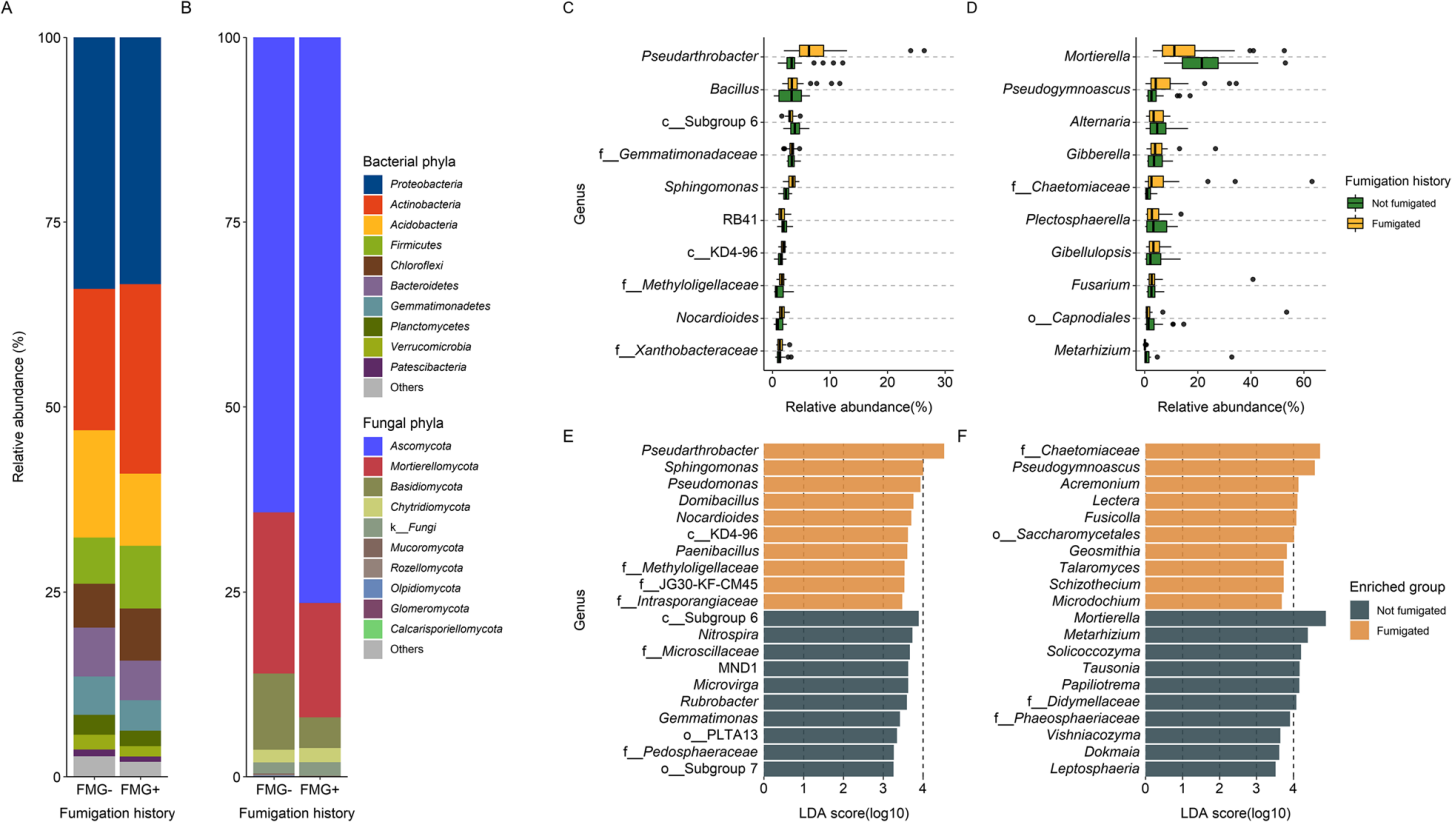
Left: Composition bar plots to show the relative abundance of the predominant bacterial (**A**) and fungal (**B**) phyla in the previously fumigated (FMG+) and non-fumigated (FMG−) soils. Top right: Box plots illustrate the ten most relatively abundant bacterial (**C**) and fungal (**D**) genera and their relative abundance between the two classifications of fumigation history. Bottom right: Log transformed Linear Discriminant Analysis (LDA) score of the top 10 enriched bacterial (**E**) and fungal (**F**) genera identified by LEfSe analysis in the previously fumigated and non-fumigated soils. A prefix indicates an unknown genus and its higher taxonomy rank was used instead, with “k_” representing “Kingdom”, “p_” for “Phylum”, “c_” for “Class”, “o_” for “Order, and “f_” for “Family”

Predominant bacterial and fungal taxa were similar but varied in relative abundance between the fumigated and non-fumigated soils, depending on the taxa and fumigation history (Fig. 3). Bacterial phyla *Proteobacteria*, *Actinobacteria*, *Acidobacteria*, *Firmicutes*, *Chloroflexi*, *Bacteroidetes*, and *Gemmatimonadetes* were predominant regardless of fumigation history (Fig. 3A), while the most abundant fungal phyla were *Ascomycota*, *Mortierellomycota*, and *Basidiomycota* (Fig. 3B). Among bacterial phyla present in fumigated soils, *Actinobacteria* were more abundant and *Acidobacteria* were less abundant (Fig. 3A). For fungi, the abundance of *Ascomycota* was higher in fumigated soils and *Basidiomycota* were more abundant in non-fumigated soils (Fig. 3B). At the genus level, the most abundant bacteria were *Pseudarthrobacter*, *Bacillus*, *Sphingomonas*, *Nocardioides*, RB41, and also several unknown genera from *Acidobacteria* Subgroup 6, *Chloroflexi* KD4-96, and from family *Gemmatimonadaceae*, *Xanthobacteraceae*, and *Methyloligellaceae* (Fig. 3C). Whereas, the predominant fungal genera were *Mortierella*, *Pseudogymnoascus*, *Plectosphaerella*, *Alternaria*, *Gibberella*, *Fusarium*, *Gibellulopsis*, *Metarhizium*, and two unknown genera from order *Capnodiales* and family *Chaetomiaceae* (Fig. 3D).

Similarly, the relative abundance of bacterial and fungal genera varied with fumigation history (Fig. 3E, F). For example, *Pseudarthrobacter*, *Sphingomonas*, *Pseudomonas*, *Domibacillus* and *Nocardioides* were significantly higher in the soils that were previously fumigated, whereas an unknown genus from *Acidobacteria* Subgroup 6, *Nitrospira*, and a genus from *Microscillaceae* were greater in the non-fumigated soils (Fig. 3E). Several fungal genera, such as a genus from family *Chaetomiaceae*, *Pseudogymnoascus*, *Acremonium*, *Lectera*, and *Fusicolla* were enriched in the fumigated soils, while *Mortierella*, *Metarhizium*, *Solicocozyma*, *Tausonia*, and *Papiliotrema* were significantly more abundant in the non-fumigated soils (Fig. 3F).

#### Microbial co-occurrence network comparison between the field soils with different fumigation history

Previous fumigation exposure altered soil bacterial and fungal co-occurrence networks (Additional File 2: Table S1 and Additional File 3: Fig. S5). For bacteria, the global network properties, such as the modularity, positive edge percentage, natural connectivity and average path length were higher in the networks constructed from fumigated soils than non-fumigated soils (Additional File 2: Table S1). The degree, betweenness, and closeness of the most influential nodes were also different between the two networks. The clustering similarity of the two networks as measured by the adjusted Rand index (ARI) was close to zero (ARI = 0.022) (Additional File 2: Table S1). Notably, a genus from order *Actinomarinales* was identified as hub taxon in the network constructed from non-fumigated soils (Additional File 3: Fig. S5). For fungi, the modularity and average path length were higher while the positive edge percentage was lower in the global networks built from the fumigated soils. However, these measurements were not statistically significant (Additional File 4: Table S2). The degree, betweenness, and closeness of the most influential fungal nodes differed between the two networks, and the ARI was also close to zero (ARI = -0.006). Additionally, genus *Talaromyces* was identified as the hub taxon in the network constructed from non-fumigated soils (Additional File 5: Fig. S6).

### Microbiome analysis of microcosm experiment

#### Soil microbial community diversity in response to metam sodium re-application

**Fig. 4:**
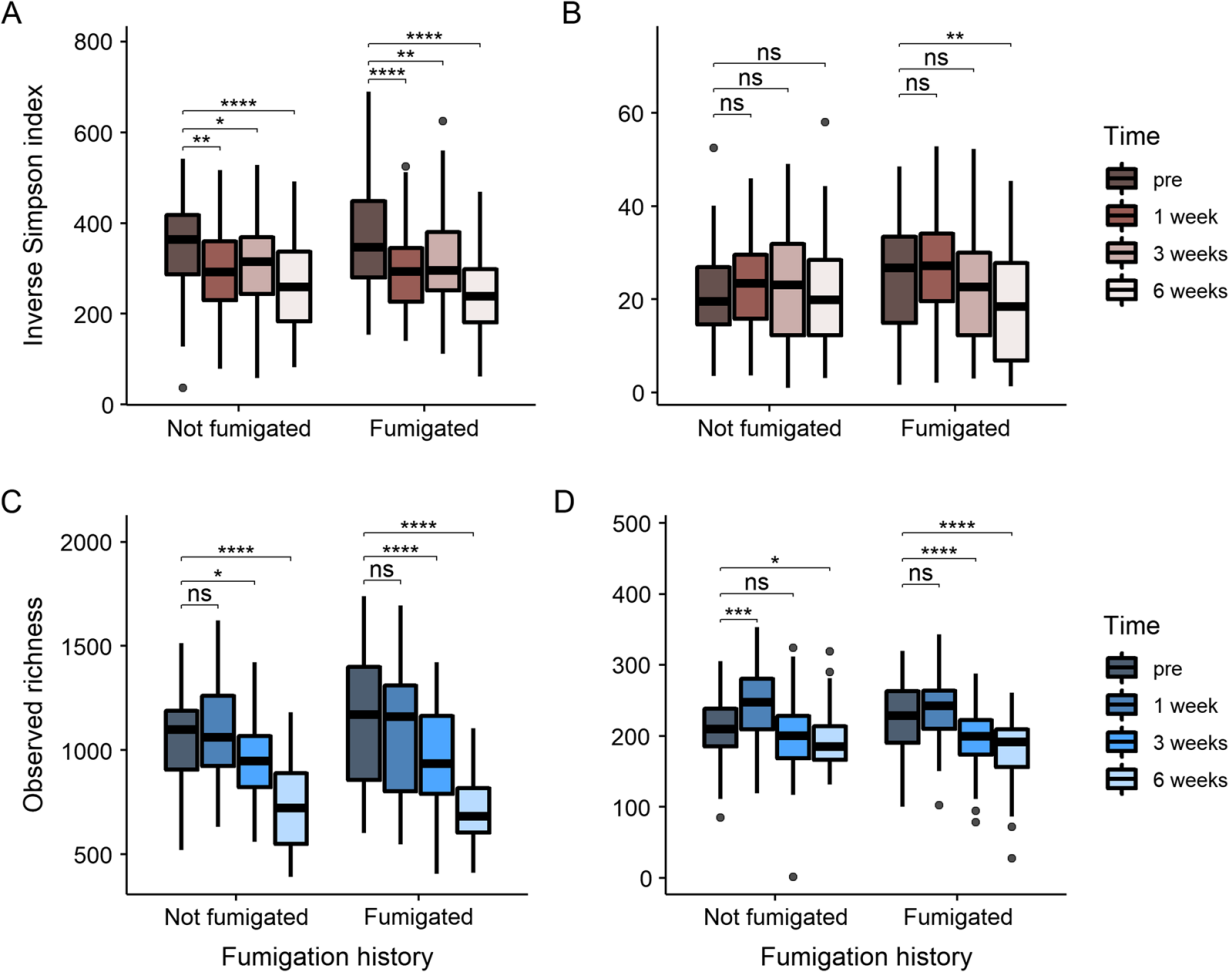
The inverse Simpson index and observed ASV richness in bacterial (**A** and **C**) and fungal (**B** and **D**) communities as a function of time after MS application and grouped by soil fumigation history. A protected t-test was used to compare diversity at each time point (i.e., 1 week, 3 weeks, and 6 weeks) to pretreatment. Significance levels were ****≤ 0.0001, ***≤ 0.001, **≤ 0.01, *< 0.05, and “ns” is not significant

Soil microbial diversity and richness changed after MS application (Table 3). In particular, bacterial diversity was decreased at one, three, and 6 weeks after MS was applied to microcosm soils (Fig. 4A), while bacterial richness was reduced three and 6 weeks after MS application (Fig. 4C). In contrast, fungal diversity of non-fumigated soils did not change after MS application but decreased in soils previous exposed to soil fumigation 6 weeks after MS application (Fig. 4B). In soils never exposed to fumigation, fungal richness increased 1 week after MS application and decreased on week six to a level that was lower than the pre-treatment richness (Fig. 4D). In the soils previously exposed to fumigation, fungal richness decreased 3 and 6 weeks after MS application (Fig. 4D).

**Table 3:** ANOVA showing the effect of fumigation history and sampling time on α-diversity in microcosm soils

Index	Factors	Bacteria		Fungi	
		*F*	*P*-value	*F*	*P*-value
Inverse Simpson index	Fumigation history	0.54	0.463	0.73	0.3942
	Time	25.84	**<0.0001**	5.74	**0.0007**
	Fumigation history × time	0.51	0.675	3.68	**0.0121**
Observed richness	Fumigation history	0.28	0.6000	0.66	0.4166
	Time	80.31	**<0.0001**	37.80	**<0.0001**
	Fumigation history × time	1.97	0.1170	2.41	0.0664

*P-*values less than 0.05 are in bold values

#### Microbial β-diversity in the microcosm experiment

Microbial β-diversity of microcosm soils was influenced by fumigation history, sampling time, soil series, rotation crop diversity, and microcosm soil pH (Additional File 1: Table S3). Specifically, fumigation history, sampling time, and soil series accounted for 4.0%, 5.0%, and 19.3% of the total variation in the bacterial β-diversity, respectively. For fungi, fumigation history, sampling time, and soil series explained 4.8%, 3.2%, and 11.1% of the variation in the fungal β-diversity. Although significant, rotation crop diversity and soil pH accounted for less than 3% of the variation in bacterial or fungal β-diversity, respectively. However, the clustering of the microbial communities of the microcosm soils resembled that of the field soils grouped by fumigation history and soil series (Figs. 2 and Additional File 1: Fig. S7). Bacterial and fungal communities of microcosm soils did not form clusters when grouped by sampling time (Additional File 1: Fig. S8A, B).

#### Predominant taxa and their relative abundance changed in response to MS treatment

**Fig. 5:**
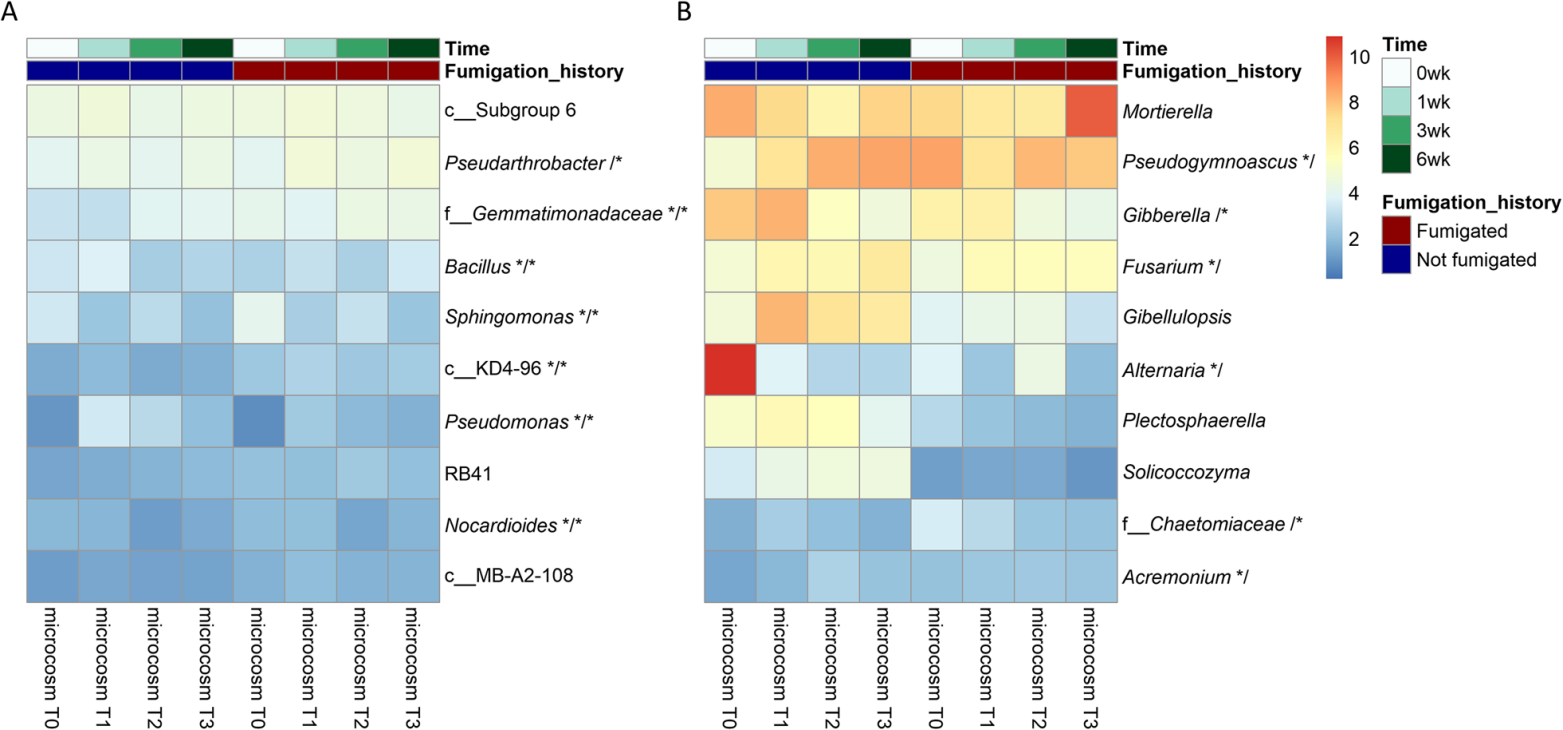
Changes in relative abundance of the top 10 predominant bacterial (**A**) and fungal (**B**) genera after metam sodium (MS) application and grouped by a soil’s previous exposure to MS and sampling time. Taxa that differed in relative abundance in time are followed by */* to indicate the taxon differed regardless of previous exposure to MS, */ to indicate the taxon only differed in soils not previously exposed to MS, and /* to indicate the taxon only differed in soils with previously exposed to MS. The color of the heatmap is scaled based on relative abundance (%) and microcosm T0 = pre-treatment (0 week), microcosm T1 = one week post treatment (1 week), microcosm T2 = 3 weeks post treatment (3 weeks), and microcosm T3 = 6 weeks post treatment (6 weeks)

The predominant bacterial and fungal genera in the microcosm soils were similar to those of the field soils, but with a few exceptions (Fig. 5). A genus from *Acidobacteria* Subgroup 6 was the most abundant after MS application, followed by *Pseudarthrobacter*, a genus of family *Gemmatimonadaceae*, *Bacillus*, *Sphingomonas*, a genus from *Chloroflexi* KD4-96, *Pseudomonas*, RB41, *Nocardioides*, and a genus from *Actinobacteria* MB-A2-108 (Fig. 5A and Additional File 1: Table S4). In contrast, fungal genera *Mortierella* and *Pseudogymnoascus* remained the top 2 most abundant, followed by *Gibberella*, *Fusarium*, *Gibellulopsis*, *Alternaria*, *Plectosphaerella*, *Solicocozyma*, a genus of family *Chaetomiaceae*, and *Acremonium* (Fig. 5B and Additional File 1: Table S5).

MS application induced variable changes in relative abundance of the predominant bacterial and fungal genera, depending on a soil’s previous fumigation history and sampling time (Fig. 5 and Additional File 1: Tables S4 and S5). For example, the relative abundance of *Bacillus* was reduced by MS application at week 3 and 6 in soils never exposed to MS but increased at week one or did not change at week 3 and 6 in soils previously fumigated (Fig. 5A and Additional File 1: Table S4). Similarly, the relative abundance of *Pseudarthrobacter* and a genus of family *Gemmatimonadaceae* was increased by MS application, but the change differed depending on a soil’s fumigation history. Additionally, several bacterial taxa demonstrated similar responses to MS treatment regardless of soil’s fumigation history. For example, the relative abundance of *Sphingomonas* was decreased and the relative abundance of *Pseudomonas* was increased at all three sampling times after MS application (Fig. 5A and Additional File 1: Table S4).

For fungi, more predominant taxa were identified as differentially abundant in soils never exposed to MS, and several of them, including *Pseudogymnoascus*, *Acremonium*, and *Fusarium*, increased in abundance 3 and/or 6 weeks after MS application (Fig. 5B and Additional File 1: Table S5). In contrast, the relative abundance of *Alternaria* was decreased at all three sampling times after MS application. However, from soils with previous MS exposure only two fungal taxa were significantly affected by MS application; the relative abundance of *Gibberella* was decreased at week three and six, while the relative abundance of a *Chaetomiaceae* genus was increased at week one (Fig. 5B and Additional File 1: Table S5).

## Discussion

Soil microbial communities are considered essential for maintaining soil health and promoting healthy plant growth [75–77], but agricultural intensification and modern agriculture management practices can have lasting effects on the soil microbiome [5, 17, 78]. In this study, the legacy effects of metam sodium (MS) on soil microbial communities and their response to MS application were examined. We found that the impacts of previous MS exposure were more pronounced on bacterial diversity, β-diversity, and co-occurrence networks, although fungal β-diversity and networks were also affected. Soil microbial composition was shifted based on fumigation history, but the change varied by taxa. In microcosms, MS application adversely affected soil microbial richness and bacterial diversity. The relative abundance of several dominant bacterial and fungal genera differentially changed after MS application, depending on a soil’s previous MS exposure, but soil microbial β-diversity remained similar to the β-diversity of the field soils that were used to establish the microcosms.

We found that bacterial diversity was lower in soils that were previously fumigated with MS, but bacterial richness was similar to that of the non-fumigated soils. This suggests bacterial evenness was changed in soils with repeated MS exposure and may be due to different sensitivities of bacteria to the active compound methyl isothiocyanate (MITC) [30, 35, 79, 80]. On the other hand, microbial richness and fungal diversity in the fumigated soils were comparable to those in the non-fumigated soils. In this study, management practices occurring on both fumigated and non-fumigated fields (e.g., incorporation of cover crops and crop residues, fertility amendments, etc.) could alleviate some of the harmful effects of fumigation on soil microorganisms [81–83]. Additionally, soil fungal communities may be more stable to repeated MS perturbation in field conditions. For one, soil fungi can decompose organic matter easily and rapidly, an advantage in competing for the soil niche space made available by fumigation [84]. Furthermore, some fungi have adopted complex mechanisms to cope with isothiocyanates, including upregulating stress and defense genes or have specific metabolic pathways to detoxify isothiocyanate [85]. However, the impacts of fumigation on soil microbial richness and diversity could also be confounded by other abiotic factors or management history associated with each individual field, such as variation in soil type [86], soil pH [87], and crop rotation [88], as bacterial diversity and richness varies greatly among fields.

Previous exposure to MS fumigation described a significant amount of variation in soil microbial β-diversity. This result is consistent with previous studies that have assessed the impacts of MS, dazomet, or allyl isothiocyanate on soil microbiota [16, 34, 35, 80, 89, 90]. Although those studies were conducted over short periods of time, there is evidence that long-term agrochemical application can change soil microbial β-diversity and alter soil processes or functions [91–93]. In addition, soil type (i.e., soil series), and diversity of previous crops grown in the fields influenced soil microbial β-diversity. In other studies, soil properties such as pH, texture, or chemical content have been shown to drive variation in soil microbial assembly, and differential abiotic conditions are likely preferred by soil microbes or microbial consortia [86, 94–96]. Crop rotation is a common agronomic practice to increase yield and profit [97]. Although there may be multiple mechanisms responsible for observed increases in yield associated with crop rotation, increased crop diversity is associated with changes to soil microbial composition that may play an important role in a soil’s disease suppressive activity [98].

The effect of MS fumigation on soil microbial composition is also evidenced by the change in relative abundance of bacterial and fungal taxa depending on fumigation history. For bacteria, *Actinobacteria* and *Firmicutes* were more abundant while *Acidobacteria* were less abundant in soils exposed to MS, this is consistent with previous studies [30, 35, 79]. At the genus level, MS fumigation exposure enriched the abundance of *Pseudarthrobacter*, *Sphingomonas*, *Pseudomonas*, *Domibacillus*, *Nocardioides*, and *Paenibacillus*—Many members of these bacteria have known beneficial functions in promoting crop growth [99, 100], antagonizing plant pathogens [100, 101], and involving in soil nitrogen cycling [79, 102]. For fungi, genera represented by *Chaetomiaceae*, *Pseudogymnoascus*, *Acremonium*, and *Lectera* were increased with MS exposure. Increased abundance of *Chaetomiaceae* has also been documented following application of dazomet and various isothiocyanates [89, 103]. Members of *Chaetomiaceae* are ubiquitous soil saprophytic fungi capable of degrading cellulose [104, 105] and its abundance increases in compost-amended soils [106], indicating this group may be able to take advantage of soil nutrients that become available after MS application more quickly than other fungi. One the other hand, repeated MS fumigation decreased *Basidiomycota*, represented by *Tremellomycetes* yeasts, such as *Solicocozyma*, *Tausonia*, *Papiliotrema*, and *Vishniacozyma*. This suggests that these soil yeasts are sensitive to MS or the active compound MITC as documented in previous research with other pesticides [107–109]. However, how fumigation affects soil yeast communities remains understudied. Because soil yeasts play an essential role in maintaining soil structure, soil nutrient cycles, and plant growth promotion [109–111], understanding how long-term management practices might have impacts on yeasts in agriculture soils would be important for maintaining soil health and improving the sustainability of farming.

The interrelationship of soil microorganisms is complex yet central to soil functions and plant performance [112, 113]. Using network analysis, soil microbial co-occurrence patterns and community stability in response to disturbance can be examined [114, 115]. To our knowledge, this is the first study investigated the soil microbial co-occurrence in response to MS fumigation. Similar to previous studies with dazomet fumigation and other fungicides [116–120], repeated MS exposure altered the network structure in bacterial and fungal communities. Specifically, the density of the associations was reduced but the ratio of positive associations was increased with MS fumigation, indicating there might have been a shift in bacterial interrelationship from competition to cooperation for utilizing the available nutrients after MITC had been degraded [116, 121]. Network clusters can represent a group of microbes with similar or related functions [112, 122]. We found the clustering agreements in the bacterial and fungal networks were different between fumigated and non-fumigated soils, respectively, implying potential functional changes as described in previous studies with fungicides tebuconazole and boscalid [119, 120]. Due to the limitation of co-occurrence network analysis [123], additional studies are needed to further characterize the microbial interactions and their functional profiles in response to repeated MS fumigation.

MS application reduced microbial richness and bacterial diversity over a period of 6 weeks regardless of a soil’s previous fumigation exposure, suggesting MS or MITC remained suppressive to the soil microbes despite of previous MS exposure. This finding is consistent with other studies that have documented short-term reductions to soil microbial populations caused by MS application [34, 35, 124, 125]. However, we did not observe bacterial diversity and richness recover as reported by Li et al. [35] and Nicola et al. [89]. This is likely explained by the shorter length of our sampling time, or our microcosms (although an open system) did not have unlimited resources to support the microbial communities that were present. In general, soil microbial communities may be resistant or resilient to environmental perturbations, either not changing after a disturbance or returning to their original state after a period of time [126]. The mechanisms of soil microbial resilience to disturbance remain unclear, although in the case of fumigation, one explanation could be the relatively short half-life (i.e., about 7 days) of the effective compound methyl isothiocyanate (MITC) decomposed from MS or dazomet in soil [127]. Additionally, the overall generation and dissipation of MITC is a function of MITC concentration and time (C x T), which also depends on many biotic (i.e., microbial processes) and abiotic (i.e., soil types) factors in a field [27].

MS application induced varied changes to relative abundance among the predominant bacterial and fungal taxa that were dependent on a soil’s previous exposure to MS. For example, the relative abundance of *Pseudarthrobacter* increased more in soils with previous MS exposure than not exposed to MS. In contrast, the relative abundance of *Bacillus* was suppressed at week 3 and 6 in microcosm soils not previously fumigated but increased (week one) or was unchanged (weeks 3 and 6) in soils that were previously fumigated. This suggests fumigation exposure could alter microbial sensitivity to MS (or MITC) in soils. However, this change in sensitivity was more apparent among fungi, as fewer dominant fungal taxa responded to MS application in soils with previous MS exposure when compared to soils with no MS exposure. Interestingly, two genera containing several plant pathogens [128, 129] varied differentially depending on previous exposure to fumigation. The relative abundance of *Alternaria* declined significantly following MS application in soils with no previous MS exposure, while its abundance did not change in soils with previous MS exposure. In contrast, the relative abundance of *Fusarium* was increased in soils with no previous MS exposure. The varied changes of different microbial taxa, dependent on previous exposure to fumigation, suggest the response of the soil microbiome to fumigation is complex, context dependent, and may be difficult to predict given the diversity of management practices that occur in intensively managed cropping systems.

## Conclusions

There are legacy effects on the soil microbiome associated with the use of MS, which are likely confounded by and interact with other abiotic properties and management practices. Based on the response of the soil microbiome to MS application, repeated fumigant applications may result in differential MS sensitivity among soil microbes. In the short-term, soil microbial structure appears to be robust to MS fumigation, but microbial richness and bacterial diversity are temporarily reduced after fumigation. The response of the soil microbiome to fumigation is context dependent, depending on previous cropping history, and may be difficult to predict considering the diversity of management practices that occur in intensively managed cropping systems. Additionally, managing the soil microbiome to enhance crop productivity will require knowledge of each field’s soil abiotic and biotic properties, environmental characteristics, and management history, necessitating maintenance of comprehensive and long-term management records for each (field) management unit.

## Supplementary Information

**Additional file 1**. Supplementary tables and figures.

**Additional file 2**. **Table S1**. Soil bacterial network comparison between fumigated soils and non-fumigated soils.

**Additional file 3**. **Figure S5**. Bacterial network comparison at the genus level between soils of fumigated (left) and non-fumigated (right) fields. Each node represents a bacterial genus. The size of a node is scaled by its degree centrality value and hubs are identified with black bold font and gray colored line around the circumference of the node. Network clusters or modules are grouped by color. The correlation between two nodes is represented by network edges (Turquoise=negative correlation, Orange=positive correlation). A prefix indicates an unknown genus and its higher taxonomy rank was used instead, with “k_” representing “Kingdom”, “p_” for “Phylum”, “c_” for “Class”, “o_” for “Order, and “f_” for “Family”.

**Additional file 4**. **Table S2**. Comparison of soil fungal networks constructed from fumigated soils and non-fumigated soils.

**Additional file 5**. **Figure S6**. Fungal network comparison at the genus level between soils of fumigated (left) and non-fumigated (right) fields. Each node represents a fungal genus. The size of a node is scaled by its degree centrality value and hubs are identified with black bold font and gray colored line around the circumference of the node. Network clusters or modules are grouped by color. The correlation between two nodes is represented by network edges (Turquoise=negative correlation, Orange=positive correlation). A prefix indicates an unknown genus and its higher taxonomy rank was used instead, with “k_” representing “Kingdom”, “p_” for “Phylum”, “c_” for “Class”, “o_” for “Order, and “f_” for “Family”.

## Data Availability

The raw sequencing reads for this study are deposited on the NCBI SRA database under BioPoject PRJNA688547. The data, metadata, and scripts used for the analyses are available on the github repository, [https://github.com/lixiaopi1985/Soil_microbial_communities_responding_to_metam_sodium.git]. Field coordinates can be provided upon request.

## Abbreviations

ANOVA: Analysis of variance
ARI: Adjusted rand index
ASV: Amplicon sequence variant
CAP: Canonical analysis of principal coordinates
FDR: False discovery rate
ITS: Internal transcribed spacer
LCC: Largest connected components
LEfSe: Linear discriminant analysis effect size
MS: Metam sodium
MITC: Methyl isothiocyanate
*P*-adj: Adjusted *P*-values
PCoA: Principal coordinate analysis
PERMANOVA: Permutational multivariate analysis of variance
QIIME2: Quantitative Insights into Microbial Ecology (version 2)
