# Supplementary material for: Legacy effects of fumigation on soil bacterial and fungal communities and their response to metam sodium application
Source: Environ Microbiome. 2022 Dec 3;17:59. doi: 10.1186/s40793-022-00454-w (PMC9719244; doi:10.1186/s40793-022-00454-w)
Supplement: Supplementary file 1 — Additional file 1. Supplementary tables and figures. [file 40793_2022_454_MOESM1_ESM.docx]

Legacy effects of fumigation on soil bacterial and fungal communities and their response to metam sodium application

**Authors:** Xiaoping Li^1^, Victoria Skillman^2^, Jeremiah Dung^3,4^, and Kenneth Frost^2,4*^

**Affiliations:**

^1^Virginia Tech, Hampton Roads Agricultural Research and Extension Center, Virginia Beach, VA 23455

^2^Oregon State University, Hermiston Agricultural Research and Extension Center, Hermiston, OR 97838

^3^Oregon State University, Central Oregon Agricultural Research and Extension Center, Madras, OR 97741

^4^Oregon State University, Department of Botany and Plant Pathology, Corvallis, OR 97333

^*^Corresponding author: K. Frost; E-mail: [kenneth.frost@oregonstate.edu](mailto:kenneth.frost@oregonstate.edu)

**Supplemental tables**

Table S1. Comparison of soil bacterial networks constructed from fumigated soils and non-fumigated soils **(Table is in a separate file: Additional file 2)**

Table S2. Comparison of soil fungal networks constructed from fumigated soils and non-fumigated soils **(Table is in a separate file: Additional file 4)**

Table S3. Pseudo *R*-squared, *F*-values and associated *P-*values estimated using marginal tests within a PERMANOVA, to determine the relative importance of each model term on β-diversity of bacterial and fungal communities in microcosm soils (α = 0.05). PERMANOVA was conducted on the Bray-Curtis dissimilarity distance matrix and heterogeneity of the categorical variables was assessed

| **Variable** | **Bacteria** | | | |  | **Fungi** | | | |
| --- | --- | --- | --- | --- | --- | --- | --- | --- | --- |
|  | **PERMANOVA** | | **Dispersion** | |  | **PERMANOVA** | | **Dispersion** | |
|  | ***R*^2^** | ***P*-value** | **Pseudo-*F*** | ***P*-value** |  | ***R*^2^** | ***P*-value** | **Pseudo-*F*** | ***P*-value** |
| Fumigation history | 0.040 | <0.0001 | 45.43 | <0.0001 |  | 0.048 | <0.0001 | 17.15 | <0.0001 |
| Soil series | 0.193 | <0.0001 | 12.40 | <0.0001 |  | 0.111 | <0.0001 | 19.32 | <0.0001 |
| Crop diversity | 0.023 | <0.0001 | -^a^ | - |  | 0.011 | <0.0001 | - | - |
| Soil pH | 0.017 | <0.0001 | - | - |  | 0.015 | <0.0001 | - | - |
| Sampling time | 0.050 | <0.0001 | 9.27 | <0.0001 |  | 0.032 | <0.0001 | 11.30 | <0.0001 |

^a^: Not tested

*P*-values less than 0.05 are shaded gray

Table S4. Relative abundance and changes to relative abundance (i.e., increases or decrease) of the top 10 predominant bacterial genera after MS application to microcosm soils with different exposure to fumigation

| **Genera** | **RA**^a^ | **Fumigation history – Not fumigated** | | | |  | **Fumigation history - Fumigated** | | | |
| --- | --- | --- | --- | --- | --- | --- | --- | --- | --- | --- |
|  |  | ***P*-adj** | **1 week**^b^ | **3 weeks** | **6 weeks** |  | ***P*-adj** | **1 week** | **3 weeks** | **6 weeks** |
| c__Subgroup6^c^ | 4.52 | 0.6534 |  |  |  |  | 0.3676 |  |  |  |
| *Pseudarthrobacter* | 4.32 | 0.2165 | + |  |  |  | 0.0062 | + + + |  | + + |
| f__*Gemmatimonadaceae* | 3.84 | 0.0002 |  | + + + | + + + |  | 0.0062 |  | + |  |
| *Bacillus* | 3.01 | 0.0002 |  | - - - | - |  | 0.0151 | + |  |  |
| *Sphingomonas* | 2.95 | 0.0002 | - - - | - - - | - - - |  | 0.0003 | - - - | - - - | - - - |
| c__KD4-96 | 2.08 | 0.0008 | + + + |  |  |  | 0.0067 | + + + |  |  |
| *Pseudomonas* | 1.95 | 0.0002 | + + + | + + + | + + + |  | 0.0003 | + + + | + + + | + + |
| RB41 | 1.89 | 0.2165 |  |  | + |  | 0.3676 |  |  |  |
| *Nocardioides* | 1.72 | 0.0002 |  | - - - | - - - |  | 0.0003 |  | - - - | - |
| c__MB-A2-108 | 1.57 | 0.2165 | + |  |  |  | 0.1767 | + |  |  |

^a^: Overall relative abundance

^b^: “+”=Model coefficient change direction suggests relative abundance increased compared to pre-treatment (0 week); “-”=Model coefficient change direction suggests relative abundance decreased compared to pre-treatment. Numbers of symbol indicate coefficient significance level without *P*-value correction, e.g., “+++”≤0.001, “++”≤0.01, “+”<0.05

^c^: A prefix indicates an unknown genus and its higher taxonomy rank was used instead, with “k_” representing “Kingdom”, “p_” for “Phylum”, “c_” for “Class”, “o_” for “Order, and “f_” for “Family”

*P-*adj values less than 0.05 are shaded gray

Table S5. Relative abundance and changes to relative abundance (i.e., increases or decrease) of the top 10 predominant fungal genera after MS application to microcosm soils with different exposure to fumigation

| **Genera** | **RA**^a^ | **Fumigation history – Not fumigated** | | | |  | **Fumigation history - Fumigated** | | | | | |
| --- | --- | --- | --- | --- | --- | --- | --- | --- | --- | --- | --- | --- |
|  |  | ***P-*adj** | **1 week**^b^ | **3 weeks** | **6 weeks** |  | ***P-*adj** | **1 week** | **3 weeks** | | **6 weeks** | |
| *Mortierella* | 7.52 | 0.0512 |  | - | - - |  | 0.5292 |  | |  | |  |
| *Pseudogymnoascus* | 7.52 | 0.0310 |  | + + | + + |  | 0.5292 |  | |  | |  |
| *Gibberella* | 6.12 | 0.0852 |  | - |  |  | 0.0050 |  | | - - | | - - |
| *Fusarium* | 5.55 | 0.0432 |  |  | + + |  | 0.2365 | + | |  | |  |
| *Gibellulopsis* | 5.24 | 0.1005 | + |  |  |  | 0.1317 | + | |  | |  |
| *Alternaria* | 4.11 | 0.0010 | - - - | - - - | - - - |  | 0.2126 |  | |  | | - |
| *Plectosphaerella* | 3.63 | 0.1005 |  |  |  |  | 0.0763 |  | |  | | - |
| *Solicoccozyma* | 2.64 | 0.3011 |  |  |  |  | 0.3028 |  | |  | |  |
| f__*Chaetomiaceae*^c^ | 2.43 | 0.1791 |  |  |  |  | 0.0085 | + | |  | |  |
| *Acremonium* | 2.10 | 0.0310 |  | + + | + + |  | 0.3210 |  | |  | |  |

^a^: Overall relative abundance

^b^: “+”=Model coefficient change direction suggests relative abundance increased compared to pre-treatment (0 week); “-”=Model coefficient change direction suggests relative abundance decreased compared to pre-treatment. Numbers of symbol indicate coefficient significance level without *P*-value correction, e.g., “+++”≤0.001, “++”≤0.01, “+”<0.05

^c^: A prefix indicates an unknown genus and its higher taxonomy rank was used instead, with “k_” representing “Kingdom”, “p_” for “Phylum”, “c_” for “Class”, “o_” for “Order, and “f_” for “Family”

*P-*adj values less than 0.05 are shaded gray

**Supplemental figures**

**
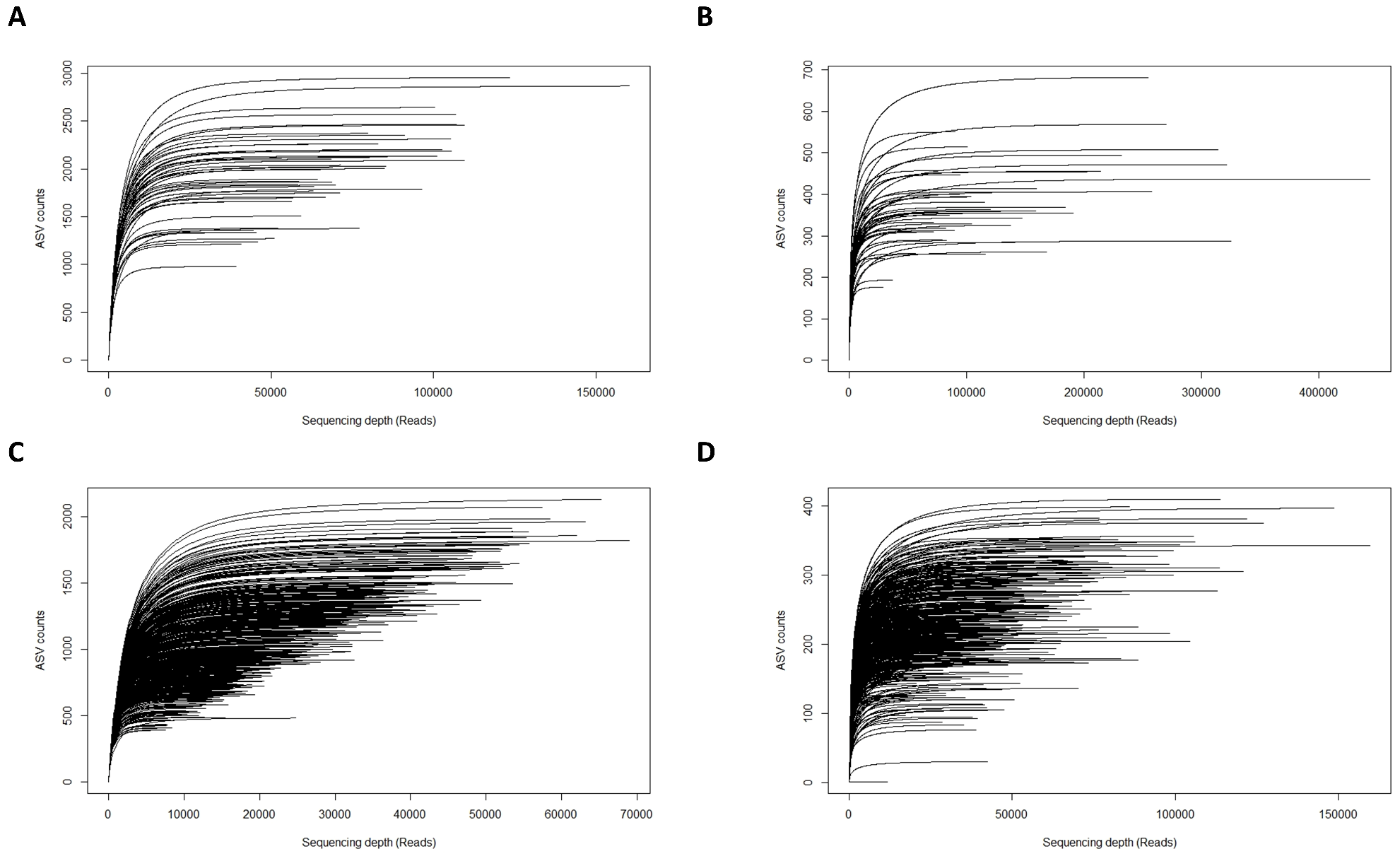
**

Figure S1. Rarefaction curves to show sequencing depth of bacterial (A) and fungal (B) communities in the field soils, and bacterial (C) and fungal (D) communities in the microcosm experiment. Each line represents a rarefaction curves of a single soil sample.


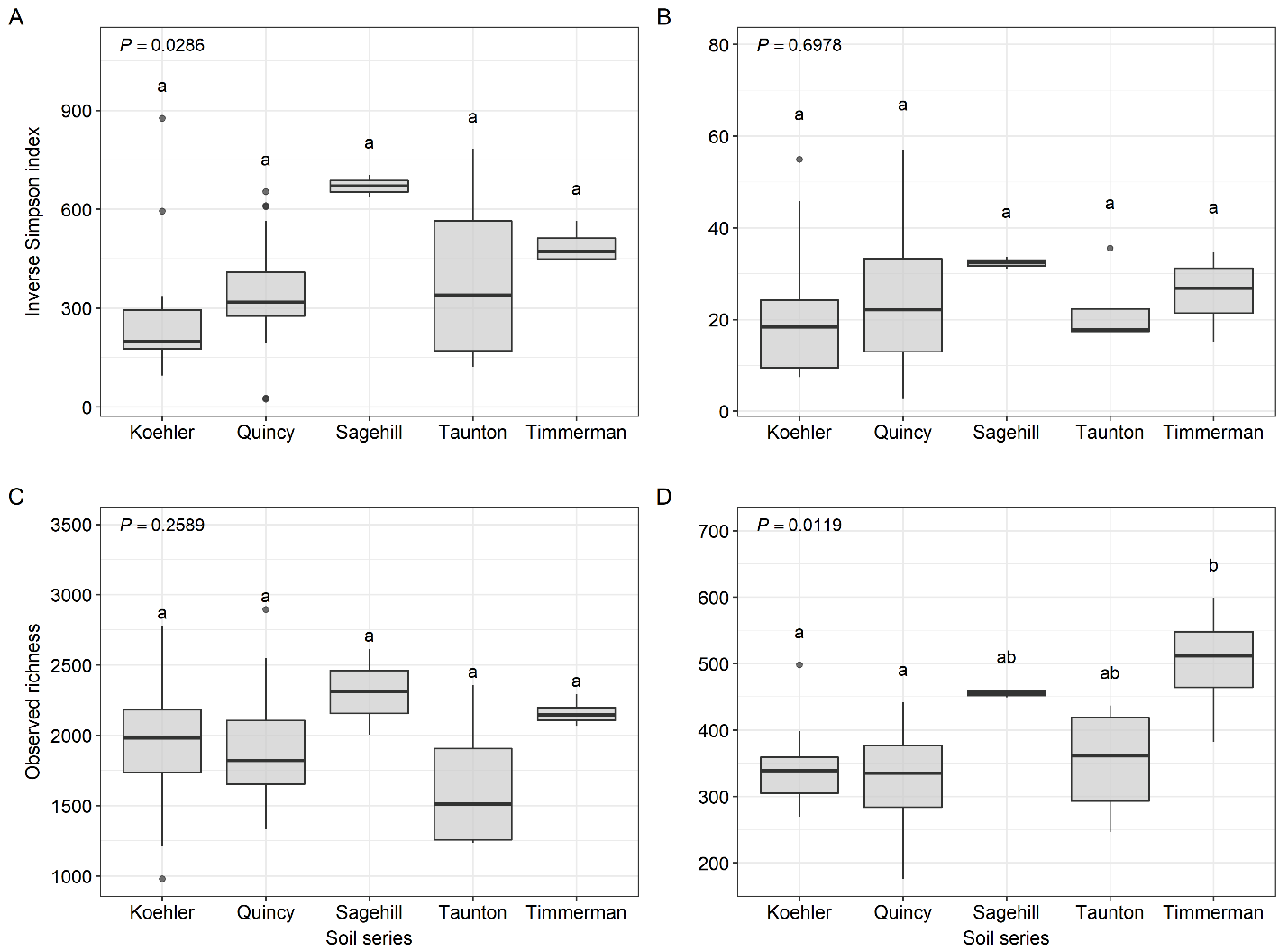


Figure S2. The inverse Simpson index and the observed ASV richness in the bacterial (A and C) and fungal (B and D) communities grouped by soil series. The overall *P-*value was calculated using the Kruskal-Wallis test. Groups with the same lower-case letter are not significantly different at α = 0.05.


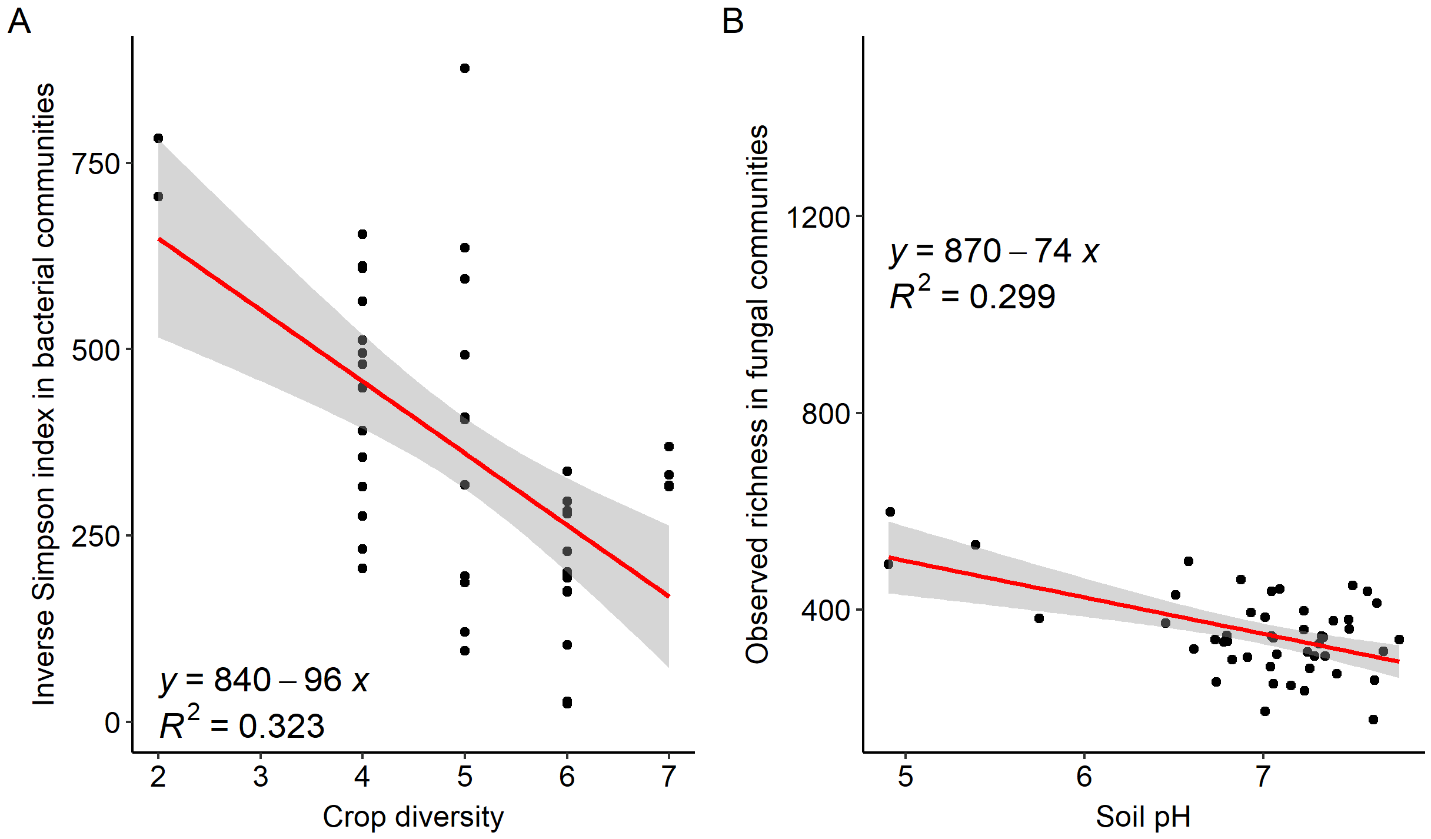


Figure S3. Linear regression to show bacterial diversity as a function of rotation crop diversity (A) and fungal richness as a function of soil pH (B).


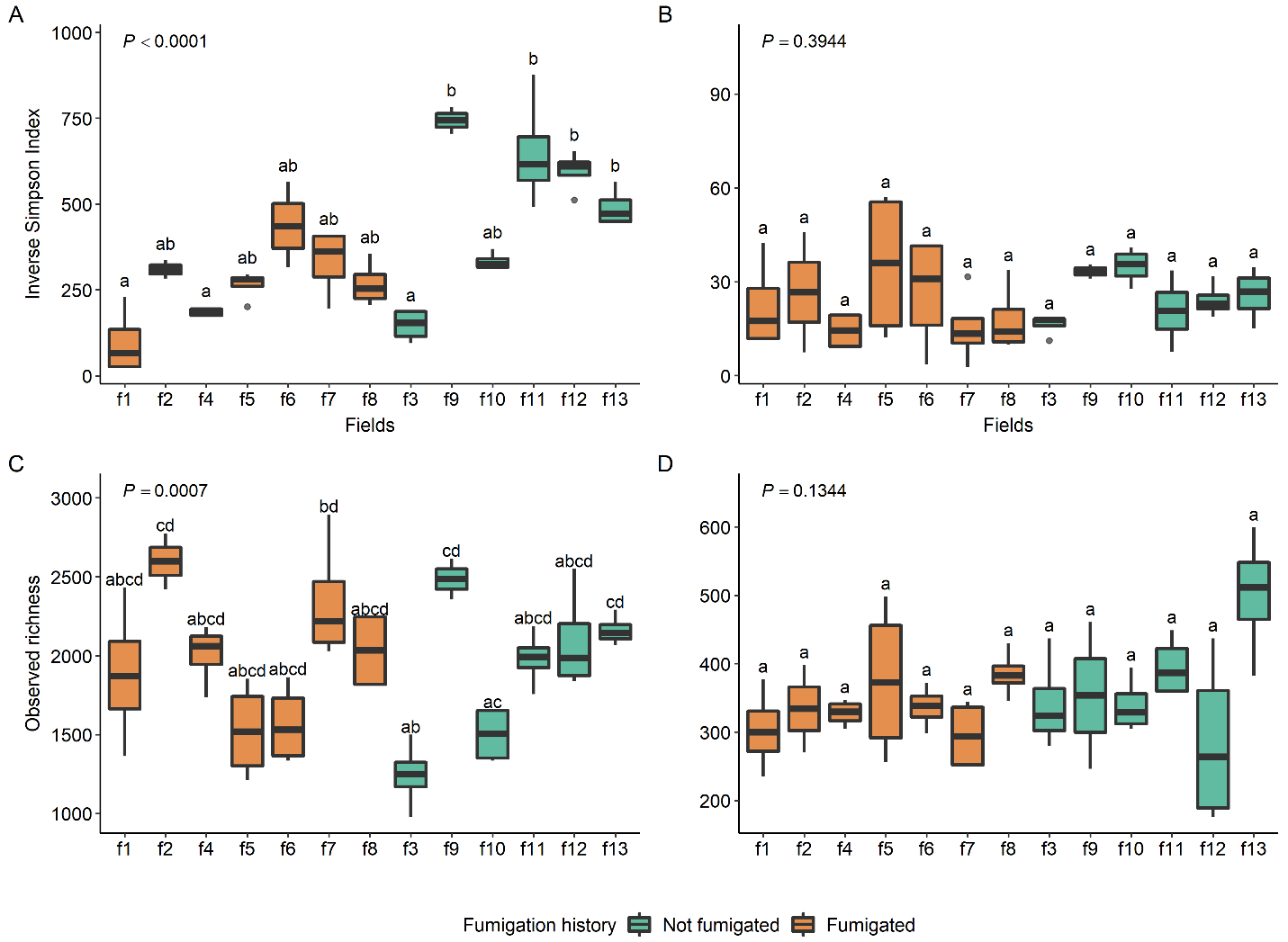


Figure S4. The inverse Simpson index and observed ASV richness in the bacterial (A and C) and fungal (B and D) communities grouped by field. Groups with the same lower-case letter are not significantly different at α = 0.05.

Figure S5. Bacterial network comparison at the genus level between soils of fumigated (left) and non-fumigated (right) fields. Each node represents a bacterial genus. The size of a node is scaled by its degree centrality value and hubs are identified with black bold font and gray colored line around the circumference of the node. Network clusters or modules are grouped by color. The correlation between two nodes is represented by network edges (Turquoise=negative correlation, Orange=positive correlation). A prefix indicates an unknown genus and its higher taxonomy rank was used instead, with “k_” representing “Kingdom”, “p_” for “Phylum”, “c_” for “Class”, “o_” for “Order, and “f_” for “Family”. **(Figure S5 is in a separate file: Additional file 3)**

Figure S6. Fungal network comparison at the genus level between soils of fumigated (left) and non-fumigated (right) fields. Each node represents a fungal genus. The size of a node is scaled by its degree centrality value and hubs are identified with black bold font and gray colored line around the circumference of the node. Network clusters or modules are grouped by color. The correlation between two nodes is represented by network edges (Turquoise=negative correlation, Orange=positive correlation). A prefix indicates an unknown genus and its higher taxonomy rank was used instead, with “k_” representing “Kingdom”, “p_” for “Phylum”, “c_” for “Class”, “o_” for “Order, and “f_” for “Family”. **(Figure S6 is in a separate file: Additional file 5)**


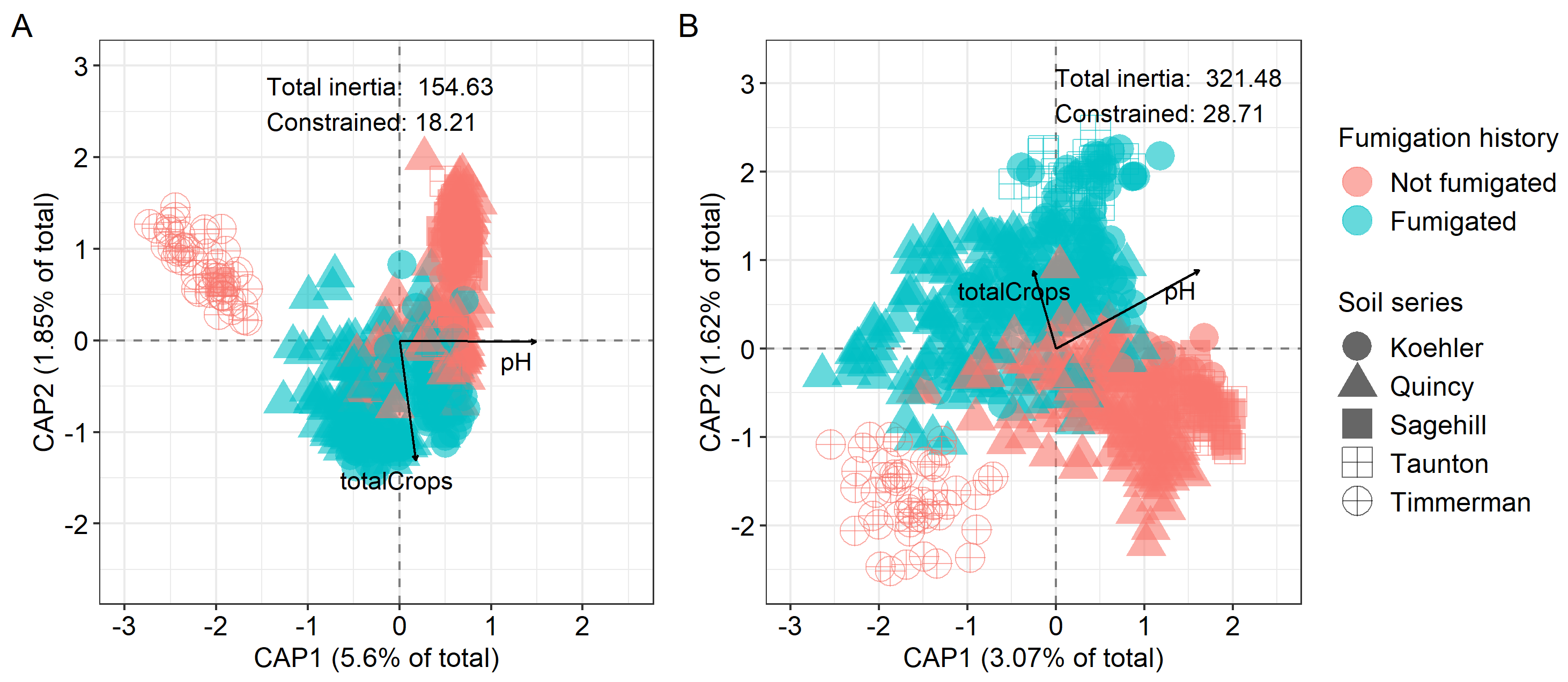


Figure S7. Bi-plot showing canonical analysis of principle coordinates (CAP) of the Bray-Curtis dissimilarity for the bacterial (A) and fungal (B) communities. Fumigation history is indecated using color and soil series is indicated using shape. Arrows represent quantitative variables that point in the direction of increase. “totalCrop” is rotation crop diversity or the total number of crops in the field management history. “pH” is soil pH.


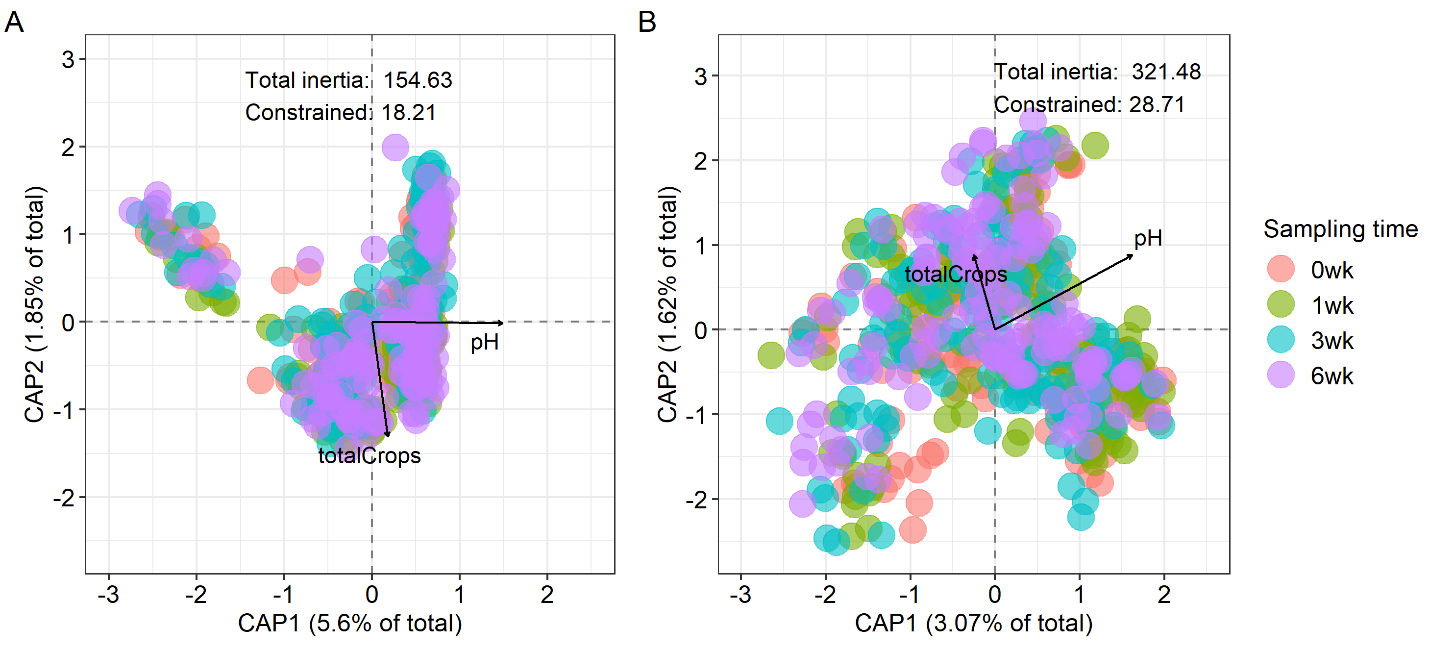


Figure S8. Bi-plot showing canonical analysis of principle coordinates (CAP) of the Bray-Curtis dissimilarity for the bacterial (A) and fungal (B) communities as a function of sampling time. Sampling time is classified by color. Arrows represent quantitative variables that point in the direction of increase. “totalCrop” is rotation crop diversity or the total number of crops in the field management history. “pH” is soil pH.
