# Supplementary figures and images for: Legacy effects of fumigation on soil bacterial and fungal communities and their response to metam sodium application
Source: Environ Microbiome. 2022 Dec 3;17:59. doi: 10.1186/s40793-022-00454-w (PMC9719244; doi:10.1186/s40793-022-00454-w)

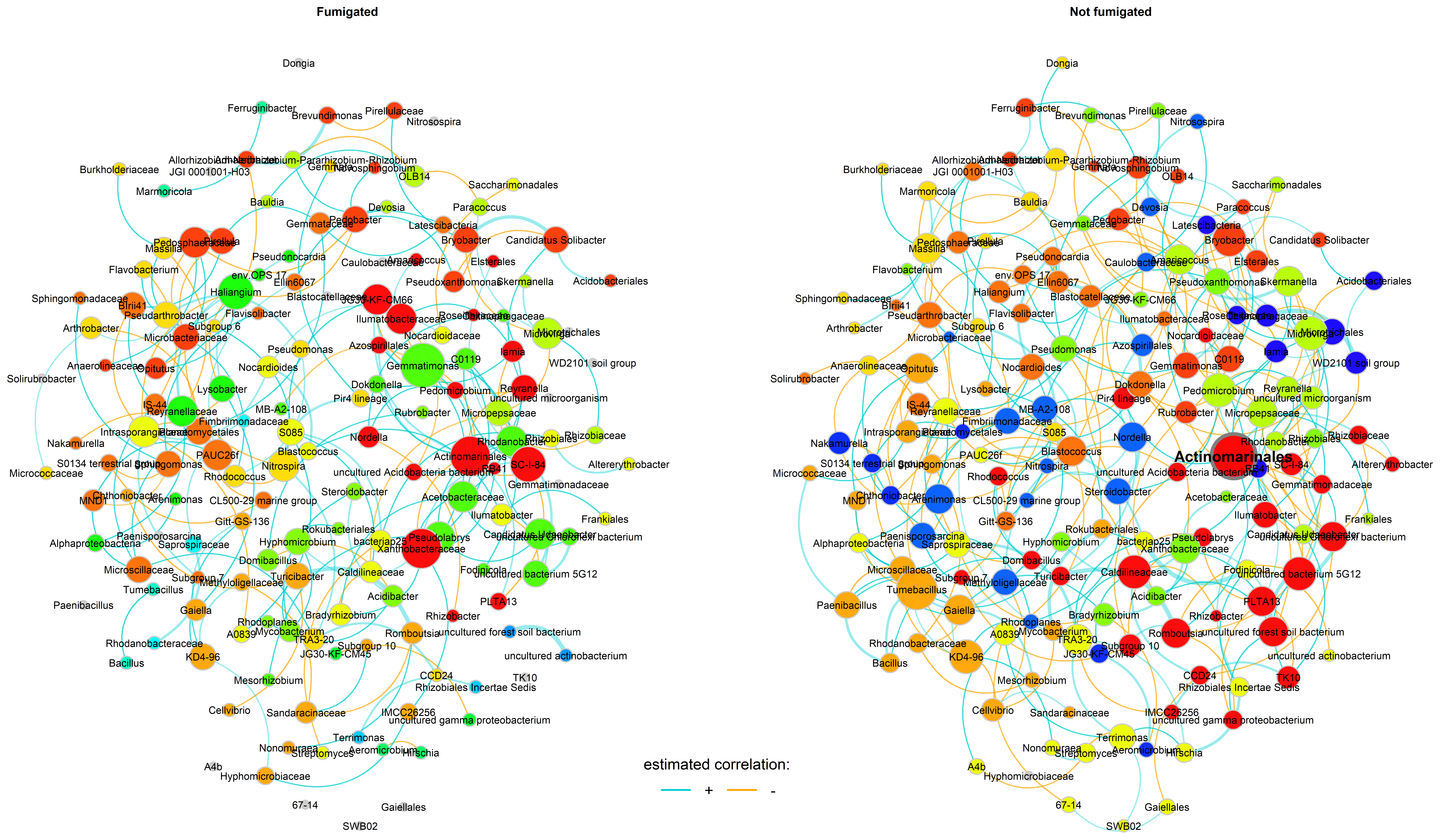

Supplement: Supplementary file 3 — Additional file 3. Figure S5. Bacterial network comparison at the genus level between soils of fumigated (left) and non-fumigated (right) fields. Each node represents a bacterial genus. The size of a node is scaled by its degree centrality value and hubs are identified with black bold font and gray colored line around the circumference of the node. Network clusters or modules are grouped by color. The correlation between two nodes is represented by network edges (Turquoise=negative correlation, Orange=positive correlation). A prefix indicates an unknown genus and its higher taxonomy rank was used instead, with “k_” representing “Kingdom”, “p_” for “Phylum”, “c_” for “Class”, “o_” for “Order, and “f_” for “Family”. [file 40793_2022_454_MOESM3_ESM.tiff]

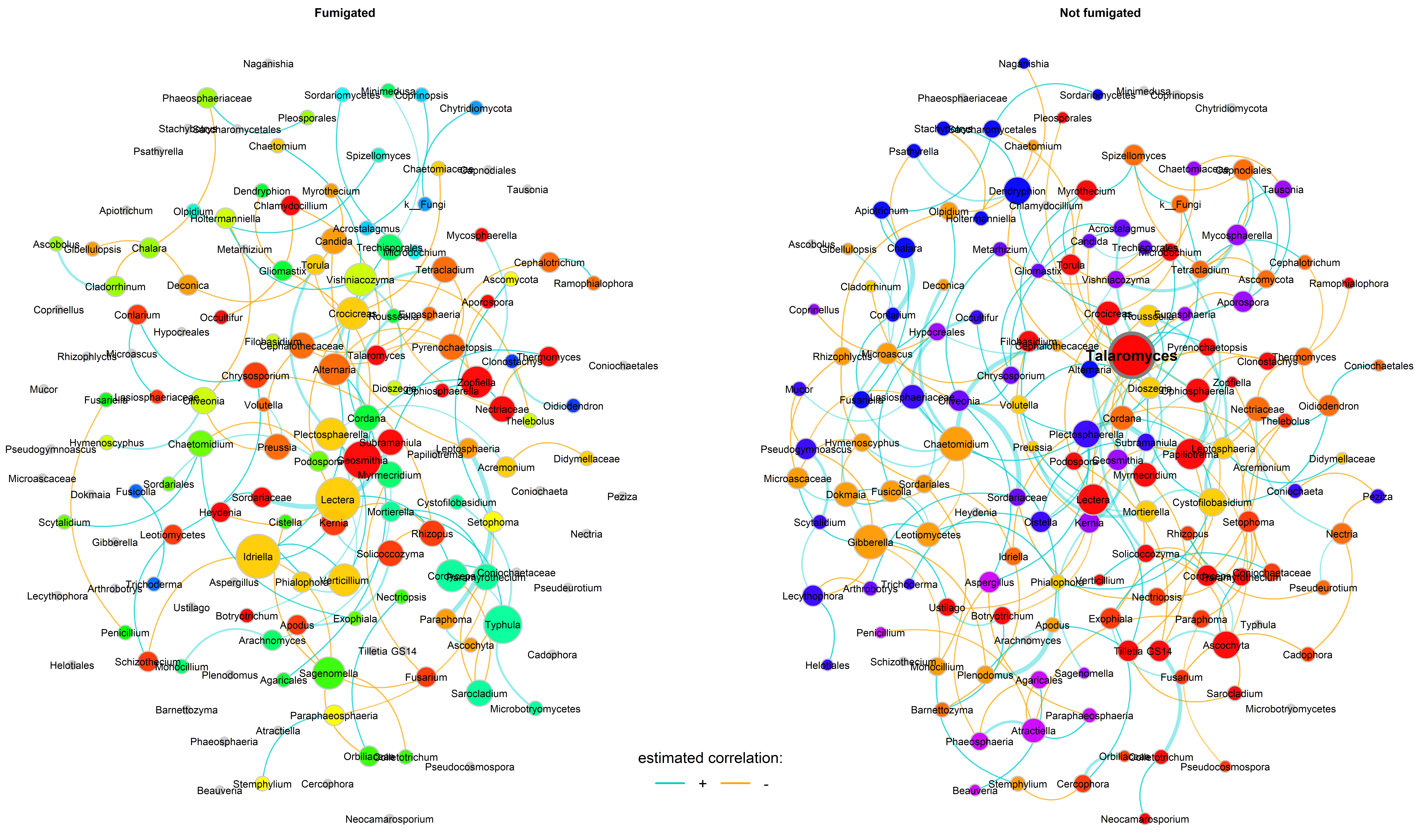

Supplement: Supplementary file 5 — Additional file 5. Figure S6. Fungal network comparison at the genus level between soils of fumigated (left) and non-fumigated (right) fields. Each node represents a fungal genus. The size of a node is scaled by its degree centrality value and hubs are identified with black bold font and gray colored line around the circumference of the node. Network clusters or modules are grouped by color. The correlation between two nodes is represented by network edges (Turquoise=negative correlation, Orange=positive correlation). A prefix indicates an unknown genus and its higher taxonomy rank was used instead, with “k_” representing “Kingdom”, “p_” for “Phylum”, “c_” for “Class”, “o_” for “Order, and “f_” for “Family”. [file 40793_2022_454_MOESM5_ESM.tiff]
